# Disease-associated mutations in inositol 1,4,5-trisphosphate receptor subunits impair channel function

**DOI:** 10.1074/jbc.RA120.015683

**Published:** 2021-01-13

**Authors:** Lara E. Terry, Kamil J. Alzayady, Amanda M. Wahl, Sundeep Malik, David I. Yule

**Affiliations:** Department of Pharmacology and Physiology, University of Rochester, Rochester, New York, USA

**Keywords:** calcium intracellular release, calcium imaging, signal transduction, calcium signaling, inositol 1,4,5-trisphosphate receptor (IP3R), spinocerebellar ataxia (SCA), Gillespie syndrome (GS), anhidrosis, calcium-intracellular release, calcium channel, inositol 1,4,5-trisphosphate (IP3), inositol trisphosphate receptor (InsP_3_R), imaging, spinocerebellar ataxia

## Abstract

The inositol 1,4,5-trisphosphate (IP_3_) receptors (IP_3_Rs), which form tetrameric channels, play pivotal roles in regulating the spatiotemporal patterns of intracellular calcium signals. Mutations in IP_3_Rs have been increasingly associated with many debilitating human diseases such as ataxia, Gillespie syndrome, and generalized anhidrosis. However, how these mutations affect IP_3_R function, and how the perturbation of as-sociated calcium signals contribute to the pathogenesis and severity of these diseases remains largely uncharacterized. Moreover, many of these diseases occur as the result of autosomal dominant inheritance, suggesting that WT and mutant subunits associate in heterotetrameric channels. How the in-corporation of different numbers of mutant subunits within the tetrameric channels affects its activities and results in different disease phenotypes is also unclear. In this report, we investigated representative disease-associated missense mutations to determine their effects on IP_3_R channel activity. Additionally, we designed concatenated IP_3_R constructs to create tetrameric channels with a predefined subunit composition to explore the functionality of heteromeric channels. Using calcium imaging techniques to assess IP_3_R channel function, we observed that all the mutations studied resulted in severely attenuated Ca^2+^ release when expressed as homotetramers. However, some heterotetramers retained varied degrees of function dependent on the composition of the tetramer. Our findings suggest that the effect of mutations depends on the location of the mutation in the IP_3_R structure, as well as on the stoichiometry of mutant subunits assembled within the tetrameric channel. These studies provide insight into the pathogenesis and penetrance of these devastating human diseases.

Inositol 1,4,5-trisphosphate (IP_3_) receptor (IP_3_R)-mediated calcium (Ca^2+^) release regulates many cellular activities including proliferation, secretion, division, contraction, and even apoptosis (1, 2, 3, 4, 5, 6, 7, 8, 9, 10, 11). There are three IP_3_R genes in the human genome (*ITPR1, ITPR2,* and *ITPR3*). The resulting monomeric isoforms of IP_3_R (IP_3_R1, IP_3_R2, and IP_3_R3) share ∼60–70% sequence homology and form homo- and heterotetrameric Ca^2+^ channels predominantly located in membranes of intracellular Ca^2+^ stores (3, 12, 13, 14, 15, 16, 17, 18). In response to various extracellular stimuli that induce phospholipase C-mediated hydrolysis of phosphatidylinositol 4,5-bisphosphate, IP_3_ is generated and diffuses through the cytosol to bind and subsequently activate IP_3_R channels resulting in movement of Ca^2+^ down its concentration gradient into the cytosol (19, 20). Each monomeric IP_3_R isoform is a ∼300 kDa protein and is generally divided into four functional domains (Fig. 1). The N-terminal suppressor domain (SD; 1-225 aa)^1^ (21) and ligand-binding domain (LBD; 226-578 aa) (22), the central regulatory and coupling domain (579-2232 aa) (23), and the C-terminal channel domain containing six membrane spanning regions and a cytosolic tail (2233-2710 aa) (24, 25, 26). It is believed that IP_3_ binding to the N terminus of IP_3_R triggers conformational changes that are transmitted intramolecularly or intermolecularly to open the channel pore located at the C terminus (27, 28, 29, 30); however, the precise mechanism of this gating is uncertain (31).

**Figure 1:**
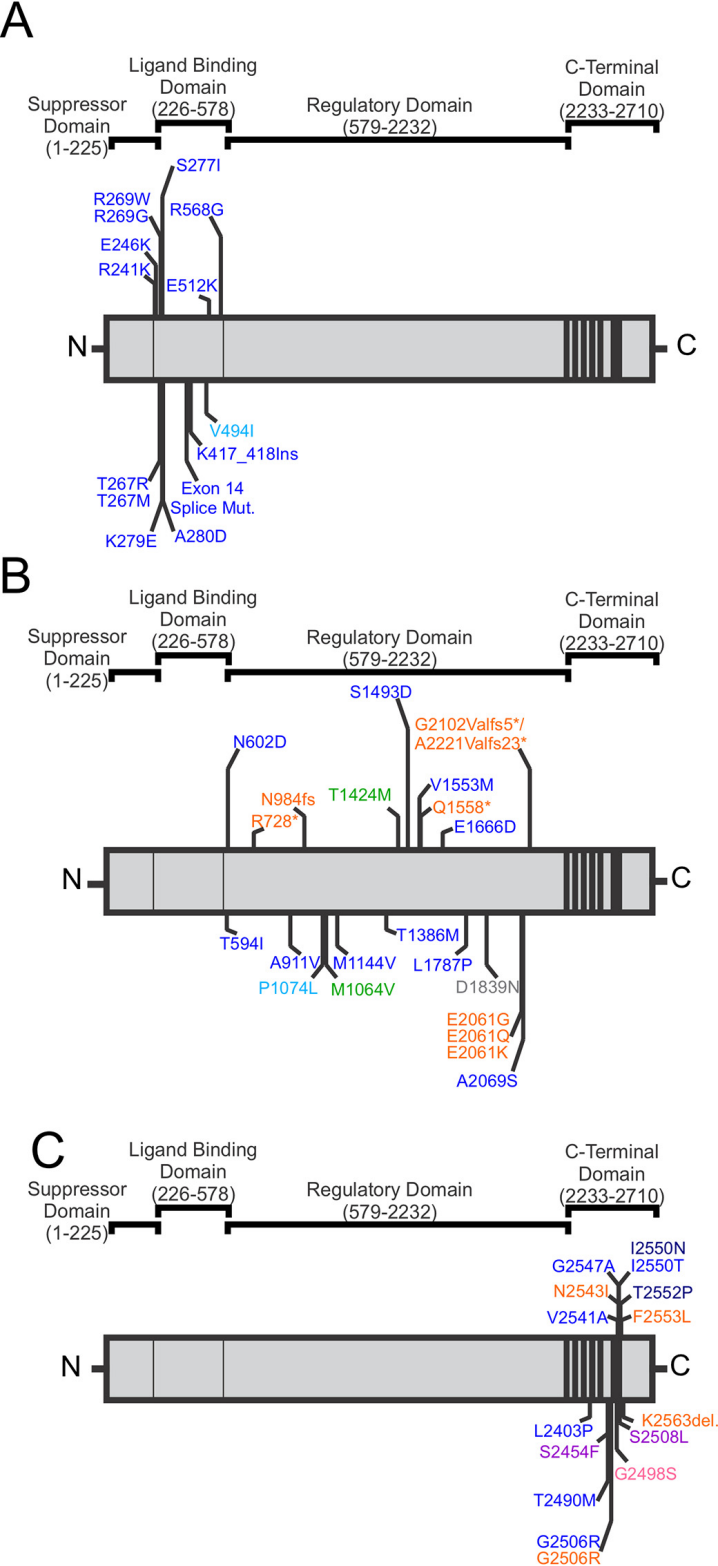
**Linear schematic of the four domains of the IP_3_R and the disease-associated IP_3_R mutations found in the LBD, regulatory and coupling, and C-terminal domains.***A,* disease-associated mutations found in the LBD (49,50, 51, 52,53, 68,69, 70,71). *B*, disease-associated mutations found in the regulatory and coupling domain (47–49, 52–54, 65, 72,73, 74, 75, 76, 77, 78, 79, 80, 81,82). *C,* disease-associated mutations found in the C-terminal domain (52,53, 54,55, 65, 83,84, 85, 86, 87, 88,89). Diseases associated with mutations in at least one isoform of IP_3_R include SCA29 (*blue*), Gillespie syndrome (*orange*), anhidrosis (*pink*), neuropathy (*green*), pontocerebellar hypoplasia (*dark blue*), spinocerebellar ataxia 15/16 (*light blue*), head and neck squamous cell carcinoma (*red*), Sézary Syndrome (*purple*), and familial isolated primary hyperparathyroidism (*gray*).

IP_3_R genes are ubiquitously expressed, although various tissues and cell types often preferentially express one subtype or a combination of subtypes (32, 33, 34, 35). Despite the overlapping expression patterns, these receptors can mediate distinct functions depending on the cell's physiological context. IP_3_R1 is highly enriched in the nervous system, whereas IP_3_R2 and IP_3_R3 are predominately expressed in the periphery including the digestive system and secretory cells of the exocrine system (36, 37, 38). Studies have shown that IP_3_R1 is essential for normal nervous system development as mice lacking IP_3_R1 often die *in utero* or suffer from severe ataxia and seizure prior to death very early in postnatal life (39, 40, 41). Mice lacking either IP_3_R2 or IP_3_R3 appear to develop only subtle phenotypes. However, mice lacking both IP_3_R2 and IP_3_R3 suffer from severe salivary and pancreatic insufficiencies and die after weaning due to inability to assimilate macronutrients from dry food (42).

Many devastating human diseases have been associated with abnormal Ca^2+^ homeostasis. Indeed, there is a growing body of literature illustrating that diseases, such as Huntington's disease, schizophrenia, ALS, Parkinson's disease, Alzheimer's disease, and spinocerebellar ataxia (SCA), are all accompanied by dysregulated Ca^2+^ signals (43, 44, 45, 46). Although the underlying etiologies of these diseases are heterogeneous, many studies have shown that perturbed IP_3_R signaling is among the contributing or causal factors. Mutations in IP_3_R genes have been associated with several human diseases, including SCA (47, 48, 49, 50, 51, 52, 53), Gillespie syndrome (GS) (52, 54), and anhidrosis (55). These mutations have generally been identified through whole exome sequencing and linkage analysis in a clinical setting. Nevertheless, the molecular and cellular consequences of many of these mutations, as well as the mechanism whereby individual mutations lead to the clinical manifestations, remain largely unknown. As mutations have been identified in all four functional domains of the IP_3_R, it is conceivable that mutations can interfere with various aspects of IP_3_R biology including IP_3_ binding, allosteric regulation by various cellular factors, ion permeation, protein folding, stability, and subcellular localization. It is likely that in patients, tetrameric IP_3_R channels are assembled from a combination of both WT and mutant subunits and given the autosomal dominant inheritance of the majority of cases, the incorporation of mutant subunits likely results in dominant-negative activity. Nevertheless, how the specific stoichiometry of the resultant heterotetrameric channels might affect function and lead to different clinical manifestations and disease penetrance have not been explored.

In this study, we have examined the consequences of a representative set of missense mutations on IP_3_R channel function that are illustrative of mutations in distinct functional domains of the IP_3_R and of differing modes of inheritance. Missense mutations of residues Arg-269 (R269W) (50, 51, 52, 53) and Asn-602 (N602D) (47, 48, 52) both lead to multiple cases of autosomal dominant, early-onset, nonprogressive ataxia, even though they are located in the LBD and regulatory/coupling domain, respectively (Fig. 1, *A* and *B*). In contrast, the G2498S mutation, located in the C-terminal selectivity filter of the IP_3_R2 channel pore (Fig. 1*C*), is inherited in an autosomal recessive fashion such that individuals homozygous for the mutation suffered from generalized anhidrosis, whereas their family members heterozygous for the mutation were asymptomatic for the disease (55). We utilized two heterologous expression systems, which are unambiguously IP_3_R *null* (56, 57) to directly investigate how expression of these mutant channels affect channel expression and localization, tetramer formation, IP_3_ binding, channel gating, Ca^2+^ store content, and Ca^2+^ release activities. To investigate how incorporation of WT and disease-associated mutant subunits into the assembled receptor alters channel function, we also used a concatenated subunit approach that allows for a defined subunit stoichiometry. Our data indicate that the impact of missense mutations is domain dependent and markedly impacted by the stoichiometry of incorporation into the functional heterotetrameric channel. These studies provide a framework to understand the molecular basis of human IP_3_R pathologies and offer novel insight into roles of key residues in IP_3_R channel activities.

## Results

IP_3_R1 is highly expressed in the nervous system (35) and mutations result in a broad range of disease phenotypes: ataxia alone or ataxia with cognitive difficulties, iris hypoplasia and/or cerebellar hypotrophy. Indeed, there is a growing list of missense (47, 48, 49, 50, 51, 52, 53, 54, 55), nonsense, insertion/deletion, and splice mutations (58, 59) scattered throughout the IP_3_R1 gene (Fig. 1). Nevertheless, how IP_3_R1 mutations affect channel activity and how these mutations lead to pathogenesis remains to be elucidated. Here we investigated in detail, the functional consequences of three representative mutations located in different functional domains of IP_3_R following expression of homotetrameric or defined heterotetrameric mutant channels in an IP_3_R *null* background.

### Mutation of R269W attenuates IP_3_R activity by reducing IP_3_ binding

**Figure 2:**
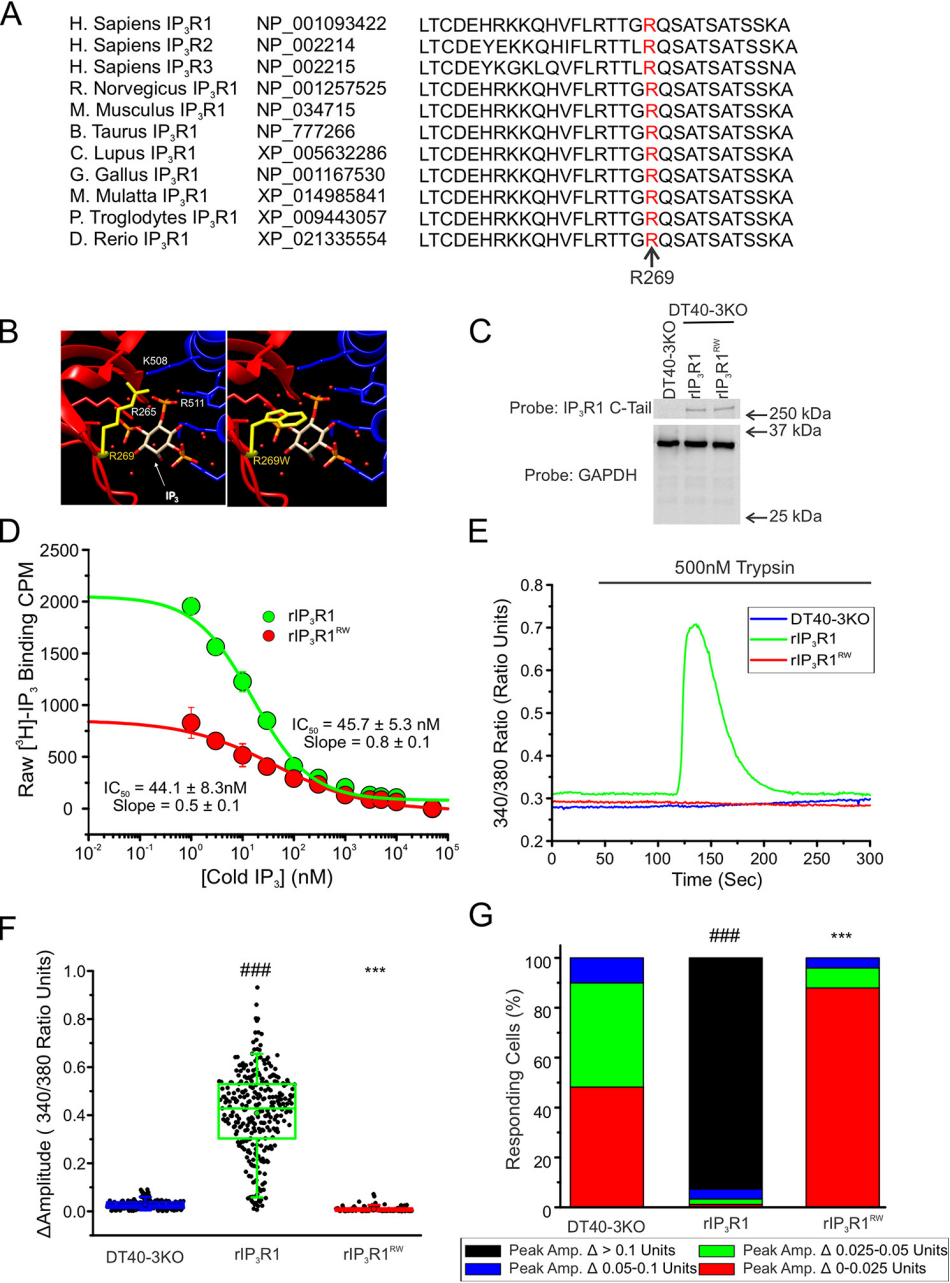
**rIP3R1RW is nonfunctional when expressed in DT40-3KO due to decreased IP3 binding.***A,* Arg-269 (*red*) is conserved among all three human IP_3_R isoforms and evolutionarily conserved in the IP_3_R1 isoform. *B,* chimera (PDB code 3JAV) was used to visualize WT Arg-269 (*yellow*, *left panel*) and R269W mutant Trp-269 (*yellow*, *right panel*) interacting with IP_3_ in the ligand binding cleft. *C,* WT rIP_3_R1 and mutant rIP_3_R1^RW^ cell lines were generated in the IP_3_R-null DT40-3KO cells and Western blotted. *D,* binding of 2.5 nm [^3^H]IP_3_ to WT rIP_3_R1 and rIP_3_R1^RW^ in the presence of increasing concentrations (0 nm, 1 nm, 3 nm, 10 nm, 30 nm, 100 nm, 300 nm, 1 μm, 3 μm, 5 μm, 10 μm, and 50 μm) of cold IP_3_ in a competitive radioligand-binding assay. Data are mean ± S.E. of three (*n* = 3) independent experiments. *E,* representative traces show Ca^2+^ signals of IP_3_R-null DT40-3KO cells (*blue*), WT rIP_3_R1 (*green*), and rIP_3_R1^RW^ (*red*) in response to trypsin (500 nm) when loaded with Fura-2/AM. *F,* scatter plots summarizing change in amplitude (peak ratio – basal ratio: average of initial 5 ratio points) for experiments similar to those shown in *E*. *Boxes* represent the 25th, 50th, and 75th percentiles, whereas whiskers represent 5th and 95th percentiles and mean is represented by *colored circles*. *G,* stacked bar graph summarizing the percentage of amplitudes from *F*, which fall into pre-determined ranges such that only those cells with an amplitude change greater than 0.1 ratio units (*black* portion of bars) are considered to be responding to the trypsin stimulus shown in *E*. Unless otherwise stated, all data above comes from at least *n* = 3 experiments. ***, *p* < 0.001 when compared with WT rIP_3_R1 cell line and ###, *p* < 0.001 when compared with DT40-3KO cell line; one-way ANOVA with Tukey's test performed in *F* (*F*_19,4699_= 753.0, *p* < 0.0001) and *G* (*F*_19,50_ = 284.6, *p* < 0.0001).

R269W has been identified in patients diagnosed with autosomal dominant, nonprogressive congenital ataxia (NPCA) (50), spinocerebellar ataxia 29 (SCA29) (52), ataxic cerebral palsy (51), and early-onset ataxia (EOA) (53). These patients exhibit nonprogressive early-onset ataxia, hypotonia, and developmental delays (Table S1). Residue Arg-269 is located in the ligand-binding core of the IP_3_R1 (Table S2) that also constitutes the IP_3_R-binding protein released with inositol 1,4,5-trisphosphate (IRBIT)-binding domain (60, 61). This mutation (R269W) substitutes an evolutionarily conserved (Fig. 2*A*), positively charged, arginine residue, with a large hydrophobic, aromatic, tryptophan residue (Fig. 2*B*). This residue has been previously identified as one of the 12 positively charged amino acids absolutely required for IP_3_ binding and early studies have demonstrated that mutation of arginine to glutamine at this site (R269Q) resulted in an 80% loss of IP_3_ binding (22, 62). We therefore investigated the consequences of the R269W mutation on IP_3_ binding. WT, rat IP_3_R1 (WT rIP_3_R1), and rat cDNA engineered to express the R269W mutation (rIP_3_R1^RW^) were stably transfected into DT40-3KO cells (56) (Fig. 2*C*). [^3^H]IP_3_ binding was measured by competitive binding assays. When exogenously expressed in DT40-3KO, rIP_3_R1^RW^ IP_3_-binding activity was severely compromised compared with WT rIP_3_R1 (Fig. 2*D*). Total specific binding was reduced by 60%, whereas the apparent EC_50_ for binding was not markedly altered under these conditions. This latter observation is surprising but may reflect severe alterations in the binding kinetics (either an alteration of on- or off-rate) such that equilibrium binding is not reached for the rIP_3_R1^RW^ mutant under the same conditions as the WT rIP_3_R1.

Next, we assessed the ability of rIP_3_R1^RW^ to support IP_3_-induced Ca^2+^ release when stably expressed in DT40-3KO cells. In single cell imaging experiments, stimulation with trypsin, which activates the Gα_q_-coupled protease-activated receptor 2 (PAR2), generates IP_3_ and results in Ca^2+^ release in cells expressing rIP_3_R1 but not in IP_3_R *null*, DT40-3KO (Fig. 2*E*) (56, 57). Similarly, whereas stimulation of cells expressing rIP_3_R1 receptors mediated robust Ca^2+^ signals, cells expressing rIP_3_R1^RW^ did not exhibit any measurable response to a maximal concentration of trypsin (Fig. 2*E*). The pooled data illustrates that despite retaining modest residual binding activity, in terms of the magnitude of signal (Fig. 2*F*), and the number of cells that respond above a defined threshold (Fig. 2*G*), rIP_3_R1^RW^ is refractory to stimulation in DT40-3KO cells. Additionally, multiple, distinct clonal rIP_3_R1^RW^ cell lines generated in DT40-3KO cells exhibited similar de-creases in amplitude (data not shown). This lack of response may be related to the requirement of each IP_3_R monomer to be occupied by IP_3_ for activation (57), which given the reduced binding, would be predicted to limit this occurrence.

In support of the idea that assemblies of mutant (binding deficient) IP_3_R1 and WT IP_3_R1 are generated in cells, we performed co-immunoprecipitation experiments in cells transfected with a mCherry-tagged rIP_3_R1 and IP_3_R1^RW^. The IP_3_R1^RW^ mutant was readily detected in samples immunoprecipitated with a mCherry antibody (Fig. S1A) and thus heterotetrameric channels containing both WT and mutant subunits were formed.

**Figure 3:**
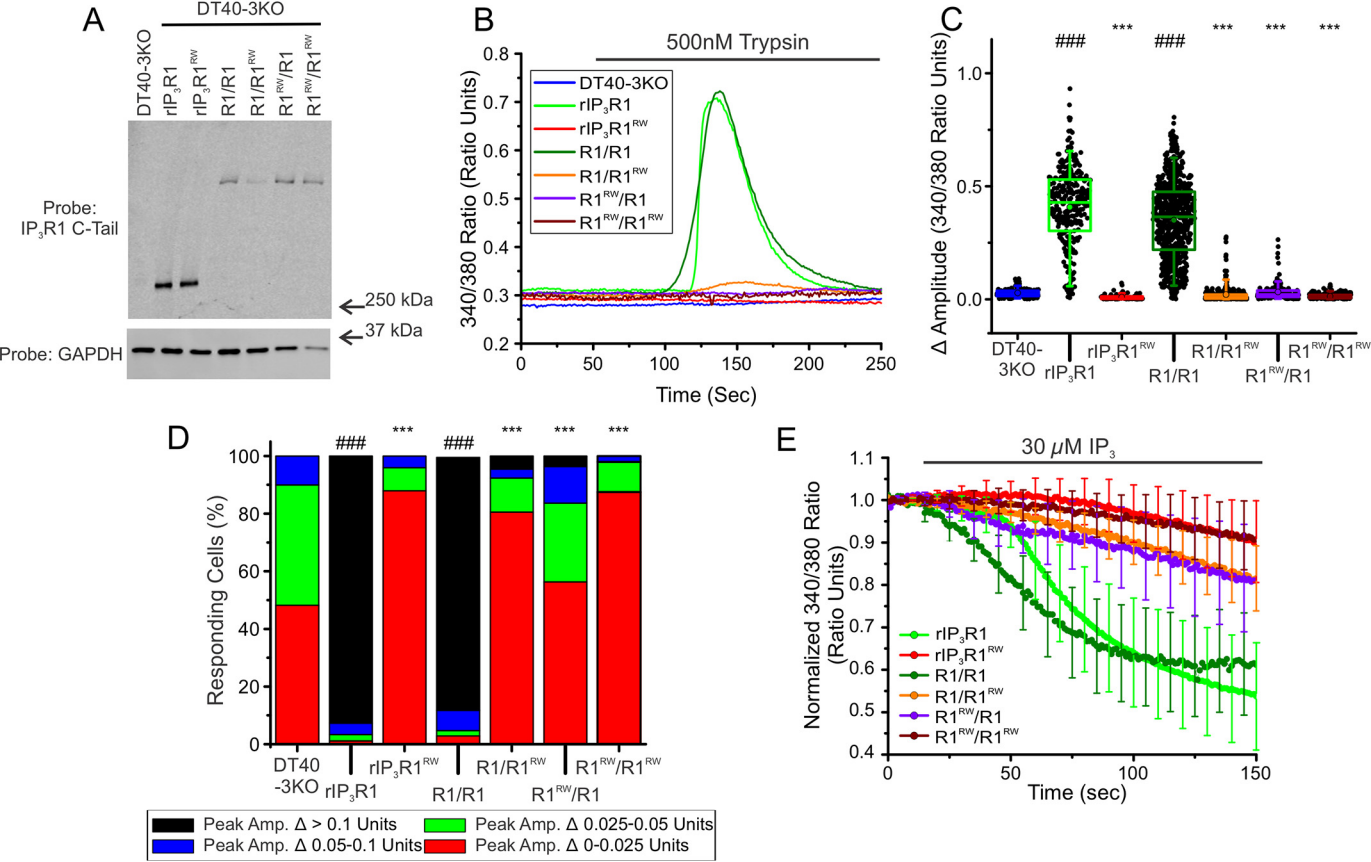
**Heterotetramers of WT rIP_3_R1 and rIP_3_R1^RW^ are predominantly nonfunctional when expressed in DT40-3KO.***A,* monomeric WT rIP_3_R1 and mutant rIP_3_R1^RW^ cell lines, as well as dimeric R1/R1, R1/R1^RW^, R1^RW^/R1, and R1^RW^/R1^RW^ cell lines were generated in the IP_3_R-null DT40-3KO cells and Western blotted. *B,* representative traces show Ca^2+^ signals of IP_3_R-null DT40-3KO cells (*blue*), WT rIP_3_R1 (*green*), and rIP_3_R1^RW^ (*red*), R1/R1 (*dark green*), R1/R1^RW^ (*orange*), R1^RW^/R1 (*purple*), and R1^RW^/R1^RW^ (*dark red*) in response to trypsin (500 nm) when loaded with Fura-2/AM. *C,* scatter plots summarizing change in amplitude (peak ratio – basal ratio: average of initial 5 ratio points) for experiments similar to those shown in *B*. *Boxes* represent the 25th, 50th, and 75th percentiles, whereas whiskers represent 5th and 95th percentiles and mean is represented by *colored circles*. *D,* stacked bar graph summarizing the percentage of amplitudes from *C,* which fall into pre-determined ranges such that only those cells with an amplitude change greater than 0.1 ratio units (*black* portion of bars) are considered to be responding to the trypsin stimulus shown in *B*. *E,* traces show Ca^2+^ signals of β-escin permeabilized WT rIP_3_R1 (*green*), and rIP_3_R1^RW^ (*red*), R1/R1 (*dark green*), R1/R1^RW^ (*orange*), R1^RW^/R1 (*purple*), and R1^RW^/R1^RW^ (*dark red*) cell lines in response to IP_3_ (30 μm) when loaded with Mag-Fura-2/AM. Data are mean ± S.E. of three (*n* = 3) independent experiments. Data for DT40-3KO, rIP_3_R1, and rIP_3_R1^RW^ in *B–D* were from Fig. 2. Unless otherwise stated, all data above comes from at least *n* = 3 experiments. ***, *p* < 0.001 when compared with WT rIP_3_R1 cell line and ###, *p* < 0.001 when compared with DT40-3KO cell line; one-way ANOVA with Tukey's test was performed in *C* (*F*_19,4699_ = 753.0, *p* < 0.0001) and *D* (*F*_19,50_ = 284.6, *p* < 0.0001).

A prediction from our previous data (57) is that heterotetramers consisting of rIP_3_R1 and rIP_3_R1^RW^ would also fail to respond to stimulation. To test this idea, we generated cDNA encoding concatenated dimers consisting of rIP_3_R1 and rIP_3_R1^RW^ expressed as a single polypeptide chain of IP_3_R1-IP_3_R1^RW^ (R1/R1^RW^) or IP_3_R1^RW^-IP_3_R1 (R1^RW^/R1) and stably expressed these constructs in DT40-3KO cells (Fig. 3*A*). Dimers are assembled by the cell to produce tetrameric channels consisting of two IP_3_R1 and two IP_3_R1^RW^ subunits. Cells expressing concatenated dimers encoding only rIP_3_R1 (R1/R1) responded to PAR2 stimulation with robust Ca^2+^ signals, as previously reported (38, 57), whereas cells expressing concatenated dimers encoding rIP_3_R1^RW^ (R1^RW^/R1^RW^) failed to respond (Fig. 3*B*). Additionally, cells expressing R1/R1^RW^ or R1^RW^/R1 dimers also failed to respond to trypsin stimulation (Fig. 3*B*). The pooled data illustrates that despite some R1/R1^RW^ or R1^RW^/R1 dimers exhibiting a very small increase in the magnitude of the signal (Fig. 3*C*), the number of cells that respond above to a defined threshold remained not significantly different from the null DT40-3KO cell line (Fig. 3*D*). Additional R1/R1^RW^, R1^RW^/R1, and R1^RW^/R1^RW^ dimeric clonal cell lines generated in DT40-3KO cells exhibited similar decreases in amplitude and percentage of cells responding above a specific threshold (data not shown). Consistent with these observations, IP_3_ also failed to evoke Ca^2+^ release in permeabilized DT40-3KO cells stably expressing channels containing two or more rIP_3_R1^RW^ subunits (Fig. 3*E*). These data indicate that tetramers containing mutant subunits likely are poorly functional. Thus, the implication is that patients with heterozygous expression of the mutant protein, only tetramers consisting of nonmutant IP_3_R1 subunits would support significant function.

**Figure 4:**
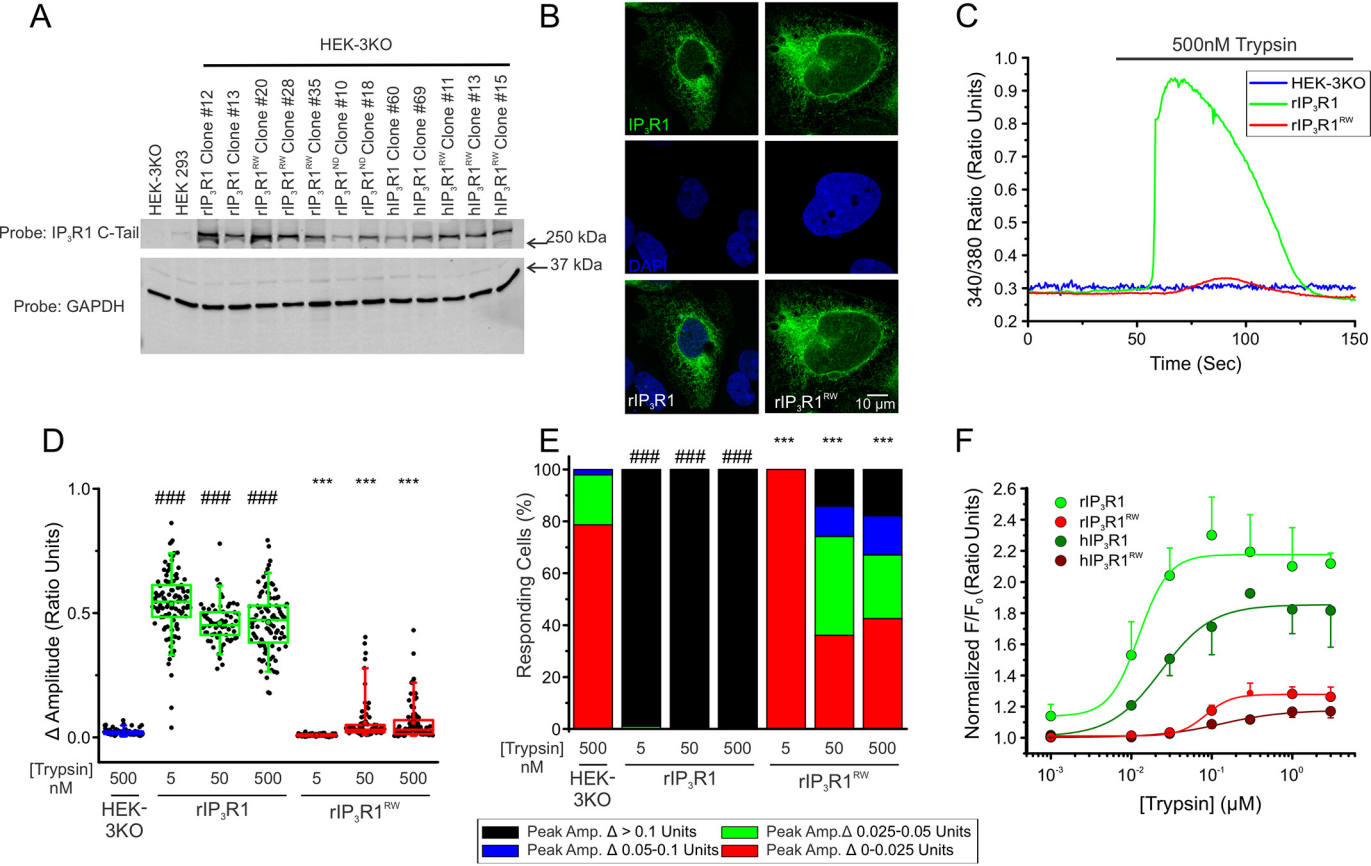
**rIP_3_R1^RW^ is poorly functional when expressed in HEK-3KO.***A,* multiple WT rIP_3_R1, WT hIP_3_R1, mutant rIP_3_R1^RW^, mutant rIP_3_R1^ND^, and mutant hIP_3_R1^RW^ cell lines were generated in the IP_3_R-null HEK-3KO cells and Western blotted. *B,* immunocytochemistry for HEK-3KO cell lines expressing either WT rIP_3_R1 (*left*) or mutant rIP_3_R1^RW^ (*right*). *Top*, IP_3_R1 detection (*green*); *middle*, DAPI detection (*blue*); *bottom*, merged images of IP_3_R1 and DAPI. *Scale bars*, 10 μm. *C,* representative traces show Ca^2+^ signals of IP_3_R-null HEK-3KO cells (*blue*), WT rIP_3_R1 (*green*), and rIP_3_R1^RW^ (*red*) in response to trypsin (500 nm) when loaded with Fura-2/AM. *D,* scatter plots summarizing change in amplitude (peak ratio – basal ratio: average of initial 5 ratio points) for experiments similar to those shown in *C* when treated with 5, 50, and 500 nm of trypsin. *Boxes* represent the 25th, 50th, and 75th percentiles, whereas whiskers represent 5th and 95th percentiles and mean is represented by *colored circles*. *E,* stacked bar graph summarizing the percentage of amplitudes from *D,* which fall into pre-determined ranges such that only those cells with an amplitude change greater than 0.1 ratio units (*black* portion of bars) are considered to be responding to the trypsin stimulus shown in *C*. *F,* dose-response curve showing Ca^2+^ response of Fura-2/AM-loaded WT rIP_3_R1, WT hIP_3_R1, rIP_3_R1^RW^, and hIP_3_R1^RW^ cells when treated with increasing concentrations (1 nm, 10 nm, 30 nm, 100 nm, 300 nm, 1 μm, and 3 μm) of trypsin using a Flexstation3 96-well–plate reader. Data are mean ± S.E. of three (*n* = 3) independent experiments. ***, *p* < 0.001 when compared with WT rIP_3_R1 cell line and ###, *p* < 0.001 when compared with HEK-3KO cell line; one-way ANOVA with Tukey's test was performed in *D* (*F*_15,1352_ = 407.1, *p* < 0.0001) and *E* (*F*_15,34_ = 108.5, *p* < 0.0001). Unless otherwise stated, all data above comes from at least *n* = 3 experiments.

Although the DT40-3KO chicken lymphocyte cell line has been used for last two decades to probe IP_3_R biology, we have recently used CRISPR/Cas9 technology to disrupt all three IP_3_R genes in human embryonic kidney (HEK293) cells to generate IP_3_R *null* HEK293 cells (HEK-3KO) (57). Tangible advantages of these cells include their mammalian origin, with presumably more relevant cellular milieu in terms of potential regulatory factors, and that HEK293 cells can be easily maintained and transfected. Therefore, we next asked if the R269W mutant behaves similarly in HEK-3KO compared with DT40 cells. Stable HEK-3KO cell lines were generated expressing WT rIP_3_R1, rIP_3_R1^RW^, or hIP_3_R1^RW^ (Fig. 4*A*). Stable expression resulted in an obvious reticular pattern of expression, not obviously different from the localization of WT IP_3_R1 (Fig. 4*B*). Expression of rIP_3_R1^RW^ did not result in alteration of the apparent content of the ER Ca^2+^ store, as assessed by the Ca^2+^ released following exposure to a SERCA pump inhibitor in a Ca^2+^-free extracellular buffer (Fig. S2, A and B). See supplemental experimental procedures. In single cell assays, stimulation of HEK-3KO expressing WT rIP_3_R1 with 5, 50, or 500 nm trypsin generated robust Ca^2+^ release (Fig. 4, *C* and *D*) with nearly 100% of cells responding (Fig. 4*E*). Notably, stimulation of cells expressing rIP_3_R1^RW^ also resulted in measurable Ca^2+^ increases (Fig. 4, *C* and *D*), albeit, at much lower maximal amplitudes and with a lower percentage of responding cells responding (Fig. 4*E*). A small increase in amplitude was likewise seen in other clonal cell lines expressing rIP_3_R1^RW^ and hIP_3_R^RW^ in HEK-3KO cells (data not shown). These findings were recapitulated in populations of cells where [Ca^2+^] changes were monitored in response to varying [trypsin] using a Flexstation3 plate reader (Fig. 4*F*). Additionally, the corresponding mutation in the human IP_3_R1 (hIP_3_R1^RW^) stably expressed in HEK-3KO cells displayed a similar marked loss of function when compared with trypsin-induced release evoked in HEK-3KO cells stably expressing hIP_3_R1 (Fig. 4*F*). The seemingly disparate findings obtained upon expression of R269W mutations in DT40-3KO and HEK-3KO could reflect differences in stable expression level of the mutant IP_3_R in HEK-3KO cells compared with DT40-3KOs (Fig. S3A). In total, these findings show in two expression systems that R269W exhibits compromised IP_3_ binding that results in severely reduced channel activity.

### Mutation of N602D attenuates IP_3_R1 activity without altering IP_3_ binding

**Figure 5:**
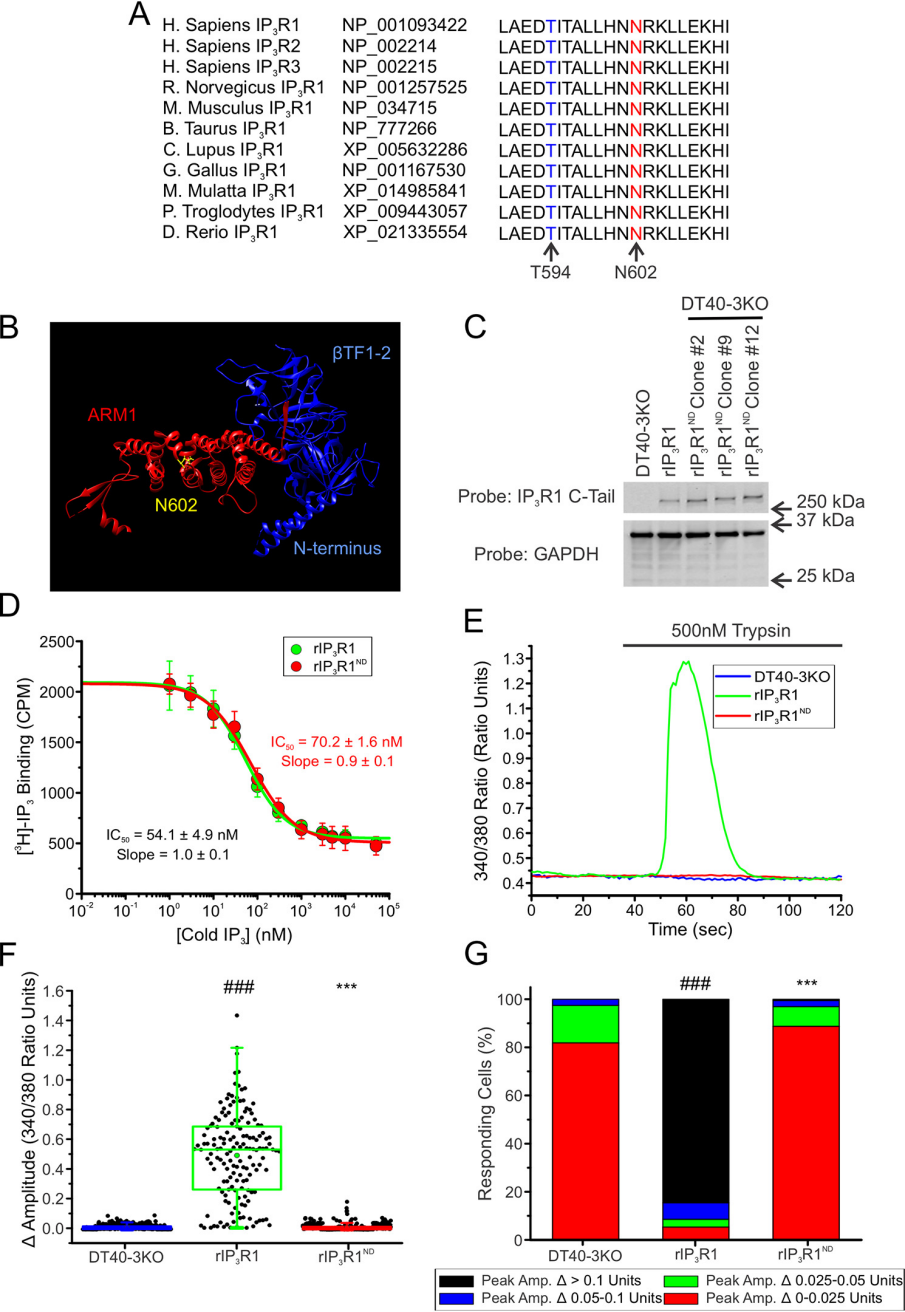
**rIP_3_R1^ND^ is nonfunctional when expressed in DT40-3KO.***A,* Asn-602 (*red*) and Thr-594 (*blue*) are conserved among all three human IP_3_R isoforms and evolutionarily conserved in the IP_3_R1 isoform. *B,* chimera (PDB 6MU1) was used to visualize WT Asn-602 (*yellow*) between two α-helical regions in the ARM1 domain (*red*) and adjacent to the β-TF1 and β-TF2 domains in the N terminus (*blue*). *C,* WT rIP_3_R1 and several mutant rIP_3_R1^ND^ cell lines generated in the IP_3_R-null DT40-3KO cells were Western blotted. *D,* binding of 2.5 nm [^3^H]IP_3_ to WT rIP_3_R1 (*green*) and rIP_3_R1^ND^ (*red*) in the presence of increasing concentrations (0 nm, 1 nm, 3 nm, 10 nm, 30 nm, 100 nm, 300 nm, 1 μm, 3 μm, 5 μm, 10 μm, and 50 μm) of cold IP_3_ in a competitive radioligand binding assay. Data are mean ± S.E. of three (*n* = 3) independent experiments. *E,* representative traces show Ca^2+^ signals of IP3R-null DT40-3KO cells (*blue*), WT rIP_3_R1 (*green*), and rIP_3_R1^ND^ (*red*) in response to trypsin (500 nm) when loaded with Fura-2/AM. *F,* scatter plots summarizing change in amplitude (peak ratio – basal ratio: average of initial 5 ratio points) for experiments similar to those shown in *E*. *Boxes* represent the 25th, 50th, and 75th percentiles, whereas whiskers represent 5th and 95th percentiles and mean is represented by *colored circles*. *G,* stacked bar graph summarizing the percentage of amplitudes from *F*, which fall into pre-determined ranges such that only those cells with an amplitude change greater than 0.1 ratio units (*black* portion of bars) are considered to be responding to the trypsin stimulus shown in *E*. Unless otherwise stated, all data above comes from at least *n* = 3 experiments. ***, *p* < 0.001 when compared with WT rIP3R1 cell line and ###, *p* < 0.001 when compared with DT40-3KO cell line; one-way ANOVA with Tukey's test was performed in F (*F*_7,3070_ = 525.9, *p* < 0.0001) and *G* (*F*_9,19_ = 177.2, *p* < 0.0001).

The N602D mutation in IP_3_R1 has been identified in two unrelated families and is associated with diagnoses of ataxic cerebral palsy without cerebral atrophy (48) and autosomal dominant congenital nonprogressive spinocerebellar ataxia (47, 52) (Table S1). Asn-602 is conserved among the three IP_3_R subtypes and across many species (Fig. 5*A*). The residue is located just outside of the LBD, but formally within the IRBIT-binding domain (Table S3) and serves to connect two α-helical regions of the receptor (Fig. 5*B*). The mutation introduces a negatively charged residue at this site, replacing an asparagine residue with a polar neutral side chain with an aspartate residue harboring a polar acidic side chain. DT40-3KO cells stably expressing WT rIP_3_R1 or rIP_3_R1 expressing N602D (rIP_3_R1^ND^) mutations were generated (Fig. 5*C*). To establish the effects of this mutation on channel function, we first assessed IP_3_ binding to the mutant proteins. In a competitive binding assay, rIP_3_R1^ND^ exhibited binding comparable with that of WT rIP_3_R1, suggesting that the mutation does not interfere with IP_3_ binding despite the proximity of the mutation to the LBD (Fig. 5*D*). To examine the consequences of the mutation on Ca^2+^ release activities, cells expressing WT rIP_3_R1 or rIP_3_R1^ND^ were stimulated with the PAR agonist, trypsin. Trypsin did not elicit any measurable [Ca^2+^] increase in cells expressing rIP_3_R1^ND^ (Fig. 5*E*). The pooled data demonstrates that in terms of the magnitude of signal (Fig. 5*F*), and the number of cells that respond above a defined threshold (Fig. 5*G*), rIP_3_R1^ND^ is refractory to stimulation in DT40-3KO cells.

**Figure 6:**
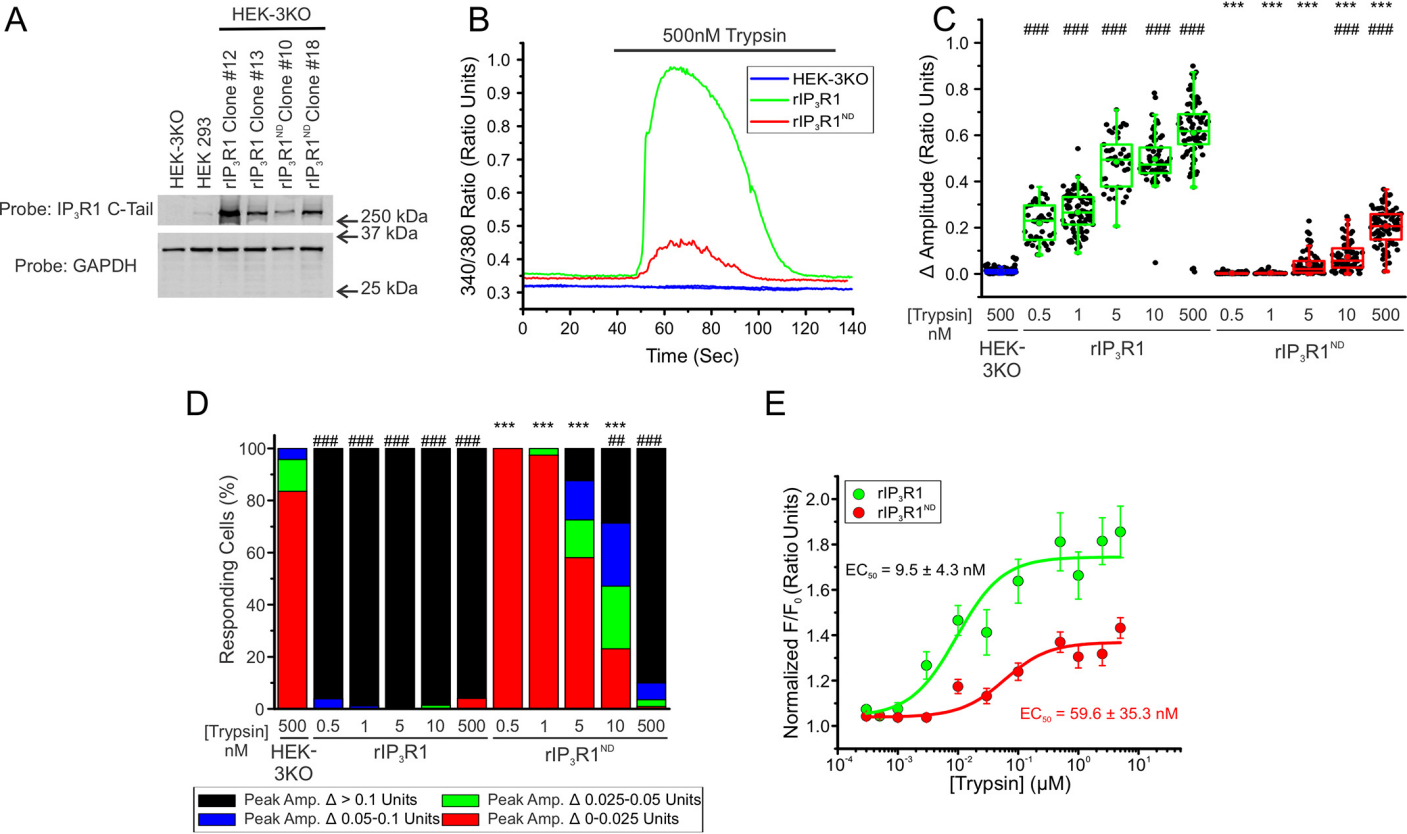
**rIP_3_R1^ND^ is poorly functional when expressed in HEK-3KO.***A,* multiple WT rIP3R1 and mutant rIP_3_R1^ND^ cell lines were generated in the IP_3_R-null HEK-3KO cells and Western blotted. *B,* representative traces show Ca^2+^ signals of IP3R-null HEK-3KO cells (*blue*), WT rIP_3_R1 (*green*), and rIP_3_R1^ND^ (*red*) in response to trypsin (500 nm) when loaded with Fura-2/AM. *C,* scatter plots summarizing change in amplitude (peak ratio – basal ratio: average of initial 5 ratio points) for experiments similar to those shown in *B* when treated with 0.5, 1, 10, and 500 nm trypsin. *Boxes* represent the 25th, 50th, and 75th percentiles, whereas whiskers represent 5th and 95th percentiles and mean is represented by *colored circles*. *D,* stacked bar graph summarizing the percentage of amplitudes from *C,* which fall into pre-determined ranges such that only those cells with an amplitude change greater than 0.1 ratio units (*black* portion of bars) are considered to be responding to the trypsin stimulus shown in *B*. *E,* dose-response curve showing Ca^2+^ response of Fura-2/AM-loaded WT rIP_3_R1 and rIP_3_R1^ND^ cells when treated with increasing concentrations (0.5 nm, 1 nm, 3 nm, 10 nm, 30 nm, 100 nm, 300 nm, 500 nm, 1 μm, 2.5 μm, and 5 μm) of trypsin using a Flexstation3 96-well–plate reader. Data are mean ± S.E. of three (*n* = 3) independent experiments. **, *p* < 0.01 and ***, *p* < 0.001 when compared with WT rIP_3_R1 cell line and ###, *p* < 0.001 when compared with HEK-3KO cell line; one-way ANOVA with Tukey's test was performed in *C* (*F*_10,791_ = 532.1, *p* < 0.0001) and *D* (*F*_10,22_ = 108.6, *p* < 0.0001). Unless otherwise stated, all data above comes from at least *n* = 3 experiments.

Given, that the rIP_3_R1^RW^ mutation exhibited more activity when stably expressed in HEK-3KO cells when compared with DT40-3KOs, we next investigated whether rIP_3_R1^ND^ was similarly more active when stably expressed in this mammalian cell line (Fig. 6*A*). Expression of rIP_3_R1^ND^ in HEK-3KO cells did not result in alteration of the apparent content of the ER Ca^2+^ store (Fig. S2C) and resulted in an obvious reticular pattern of expression (Fig. S4). Consistent, with data obtained in cells expressing rIP_3_R1^RW^, stimulation of single HEK-3KO stably expressing rIP_3_R1^ND^ with various [trypsin] resulted in modest Ca^2+^ release (Fig. 6*B*) manifested as reduced maximal initial release (Fig. 6*C*) and a lower percentage of cells responding at all [trypsin] (Fig. 6*D*). Once again, differences observed when the mutant is stably expressed in HEK-3KO cells compared with DT40-3KOs may result from differences in mutant protein expression in the two cell lines (Fig. S3B). A mutation in a neighboring residue in the same region, Thr-594 (T594I) (Fig. S5A), which is associated with infantile onset SCA (49), exhibited a similar decreased Ca^2+^ release (Fig. S5D) and percentage of cells responding (Fig. S5E) when stably expressed in HEK-3KO cells (Fig. S5B) and stimulated with a maximal concentration of trypsin (Fig. S5C). Reflecting the behavior at the single cell level, in population assays, the concentration *versus* response relationship for trypsin-induced [Ca^2+^] release reflected a decrease in both efficacy and sensitivity of stimulation (Fig. 6*E* and Fig. S5F). Thus, although competent to bind IP_3_, homomeric IP_3_R1 harboring mutations in this region are largely nonfunctional.

**Figure 7:**
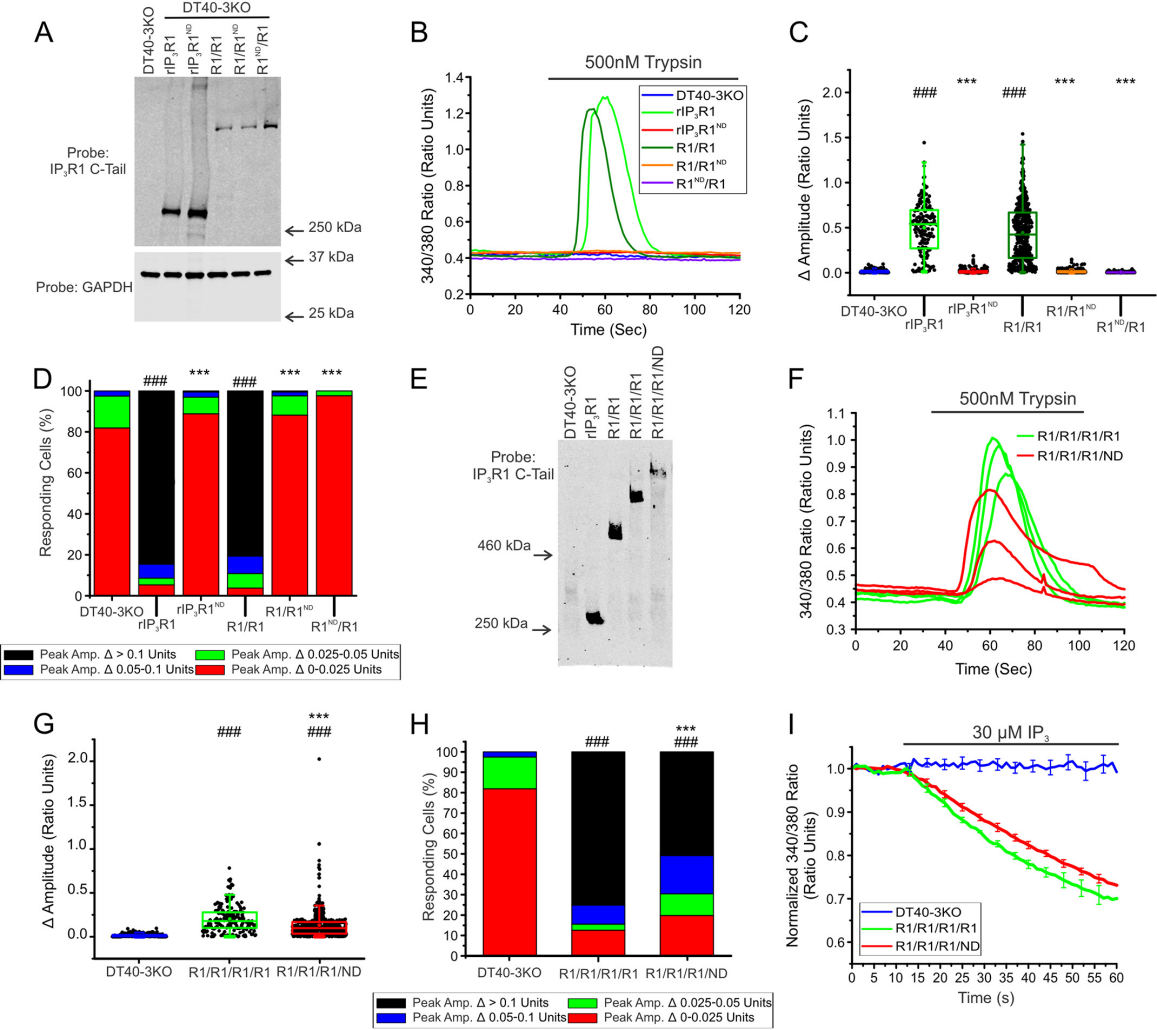
**Heterotetramers of WT rIP_3_R1 and rIP_3_R1^ND^ are predominantly nonfunctional when expressed in DT40-3KO.***A,* monomeric WT rIP_3_R1 and mutant rIP_3_R1^ND^ cell lines, as well as dimeric R1/R1, R1/R1^ND^, and R1^ND^/R1 cell lines were generated in the IP_3_R-null DT40-3KO cells and Western blotted. *B,* representative traces show Ca^2+^ signals of IP_3_R-null DT40-3KO cells (*blue*), WT rIP_3_R1 (*green*), and rIP_3_R1^ND^ (*red*), R1/R1 (*dark green*), R1/R1^ND^ (*orange*), and R1^ND^/R1 (*purple*) in response to trypsin (500 nm) when loaded with Fura-2/AM. *C,* scatter plots summarizing the change in amplitude (peak ratio – basal ratio: average of initial 5 ratio points) for experiments similar to those shown in *B*. *Boxes* represent the 25th, 50th, and 75th percentiles, whereas whiskers represent the 5th and 95th percentiles and the mean is represented by *colored circles*. *D,* stacked bar graph summarizing the percentage of amplitudes from *C*, which fall into pre-determined ranges such that only those cells with an amplitude change greater than 0.1 ratio units (*black* portion of bars) are considered to be responding to the trypsin stimulus shown in *B*. *E,* monomeric WT rIP3R1, dimeric R1/R1, trimeric R1/R1/R1, and tetrameric R1/R1/R1/ND cell lines generated in the IP3R-null DT40-3KO cells were Western blotted. *F,* multiple representative traces show Ca^2+^ signals of IP3R-null DT40-3KO cells expressing R1/R1/R1/R1 (*green*) and R1/R1/R1/ND (*red*) tetramers in response to trypsin (500 nm) when loaded with Fura-2/AM. *G,* scatter plots summarizing change in amplitude for experiments similar to those shown in *F* when treated with 500 nm trypsin. *H,* stacked bar graph summarizing the percentage of amplitudes from *G*, which fall into pre-determined ranges such that only those cells with an amplitude change greater than 0.1 ratio units (*black* portion of bars) are considered to be responding to the trypsin stimulus shown in *F*. *I,* traces show Ca^2+^ signals of β-escin permeabilized IP3R-null DT40-3KO cells (*blue*), R1/R1/R1/R1 (*green*), and R1/R1/R1/ND (*red*) cell lines in response to IP_3_ (30 μm) when loaded with Mag-Fura-2/AM. Data are mean ± S.E. of three (*n* = 3) independent experiments. Data for rIP3R1 and rIP_3_R1^ND^ in *B–D* and DT40-3KO in *B–H* came from Fig. 5. Unless otherwise stated, all data above comes from at least *n* = 3 experiments. ***, *p* < 0.001 when compared with WT rIP3R1 cell line and ###, *p* < 0.001 when compared with DT40-3KO cell line; one-way ANOVA with Tukey's test was performed in *C* (*F*_7,3070_ = 525.9, *p* < 0.0001), *D* (*F*_9,19_ = 177.2, *p* < 0.0001), *G* (*F*_7,3070_ = 525.9, *p* < 0.0001), and *H* (*F*_9,19_ = 177.2, *p* < 0.0001).

We next investigated the impact of the presence of variable numbers of mutant rIP_3_R1^ND^ within a heterotetrametric assembly of subunits, which likely represents the situation in patients heterozygous for the mutation. Once again, the IP_3_R1 protein was readily detected in samples immunoprecipitated with a mCherry antibody suggesting heterotetrameric channels containing both WT and mutant channels are formed (Fig. S1A). Concatenated dimers were generated containing one WT rIP_3_R1 and one rIP_3_R1^ND^ (rIP3R1-rIP_3_R1^ND^: R1/R1^ND^ or rIP_3_R1^ND^-rIP_3_R1: R1/R1^ND^) and stably expressed in DT40-3KO cells (Fig. 7*A*). As shown previously, tetramers expressed from either monomeric rIP_3_R1 (WT rIP_3_R1) or dimeric rIP_3_R1 (R1/R1) were capable of supporting robust PAR2-mediated Ca^2+^ signaling, however, tetramers assembled from R1^ND^/R1 or R1/R1^ND^ were completely refractory to stimulation at maximal [trypsin] (Fig. 7, *B–D*). These data indicate that tetramers harboring two mutant IP_3_R1^ND^ subunits are likely severely compromised even given the modest activity of this mutant in HEK cells. To investigate if tetramers harboring only a single mutant rIP_3_R1^ND^ retain any activity, concatenated tetramers were generated with three WT rIP_3_R1 and one rIP_3_R1^ND^ (rIP_3_R1-rIP_3_R1-rIP_3_R1-rIP_3_R1^ND^: R1/R1/R1/R1^ND^) and stably expressed in DT40-3KO cells (Fig. 7*E*). Notably, some cells expressing R1/R1/R1/R1^ND^ tetramers retained a comparable ability to support Ca^2+^ release to a concatenated tetramer assembled from WT rIP_3_R1 in terms of both trypsin (Fig. 7, *F–H*) and IP_3_-evoked Ca^2+^ release (Fig. 7*I*). In total these data demonstrate that rIP_3_R^ND^ retains modest activity and that tetramers harboring this gating mutant are not necessarily completely disabled.

### Mutation of G2498S in the channel pore attenuates IP_3_R2 activity

**Figure 8:**
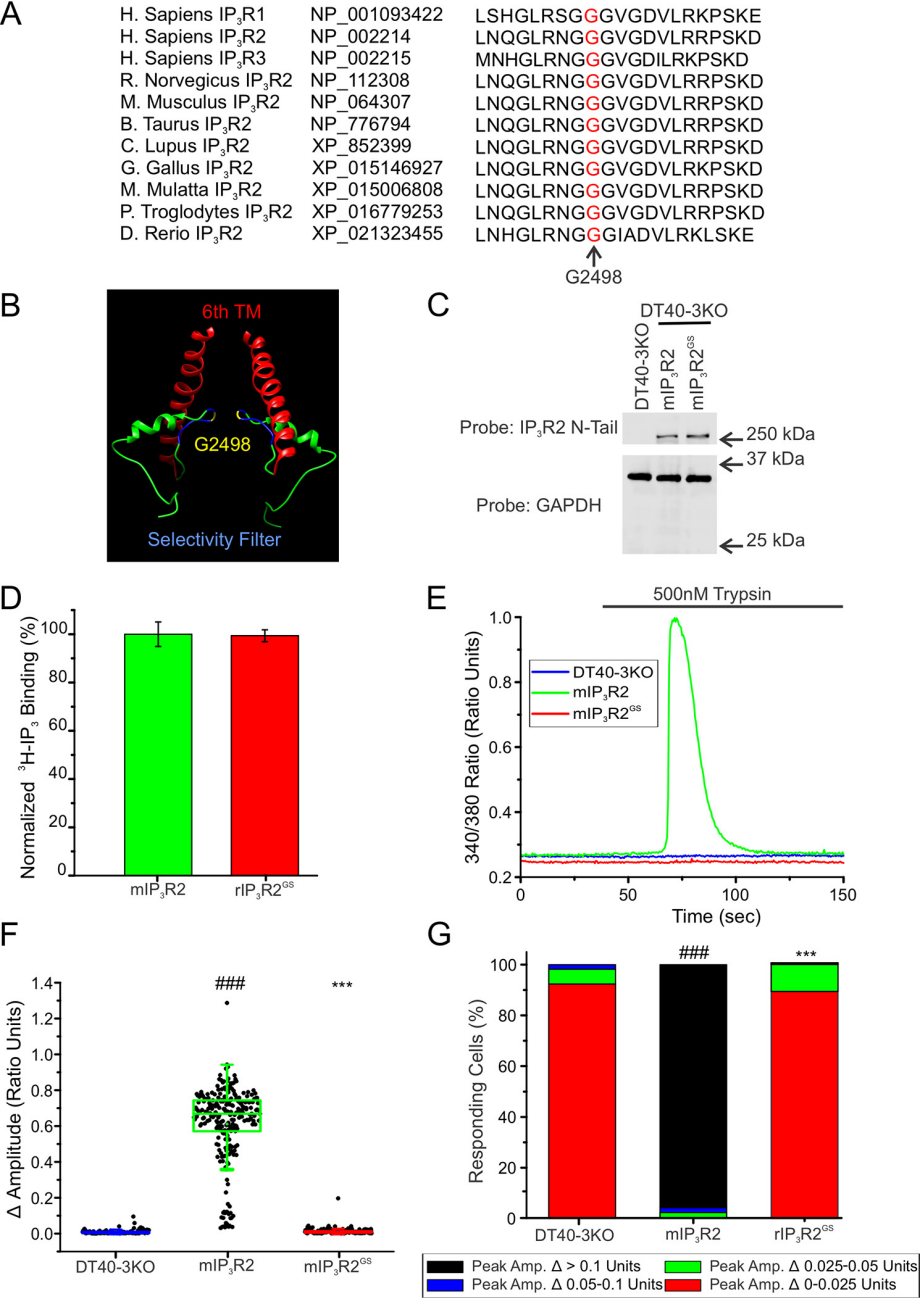
**mIP_3_R2^GS^ is nonfunctional when expressed in DT40-3KO.***A,* Gly-2498 (*red*) is conserved among all three human IP3R isoforms and evolutionarily conserved in the IP_3_R2 isoform. *B,* chimera (PDB 6MU1) was used to visualize WT Gly-2498 (*yellow*) in the selectivity filter (*blue*) of two monomers, just prior to the 6th transmembrane (*TM*) domain (*red*). *C,* WT mIP_3_R2 and mutant mIP_3_R2^GS^ cell lines were generated in the IP_3_R-null DT40-3KO cells and Western blotted. *D,* binding of 2.5 nm [^3^H]IP_3_ to WT mIP_3_R2 and mIP_3_R2^GS^ in the presence of a maximal concentration of 50 μm cold IP_3_ in a competitive radioligand binding assay (*p* = 0.9077). Data are mean ± S.E. of three (*n* = 3) independent experiments. *E,* representative traces show Ca^2+^ signals of IP3R-null DT40-3KO cells (*blue*), WT mIP_3_R2 (*green*), and mIP_3_R2^GS^ (*red*) in response to trypsin (500 nm) when loaded with Fura-2/AM. *F,* scatter plots summarizing change in amplitude (peak ratio – basal ratio: average of initial 5 ratio points) for experiments similar to those shown in *E*. *Boxes* represent the 25th, 50th, and 75th percentiles, whereas whiskers represent 5th and 95th percentiles and mean is represented by *colored circles*. *G,* stacked bar graph summarizing the percentage of amplitudes from *F,* which fall into pre-determined ranges such that only those cells with an amplitude change greater than 0.1 ratio units (*black* portion of bars) are considered to be responding to the trypsin stimulus shown in *E*. Unless otherwise stated, all data above comes from at least *n* = 3 experiments. ***, *p* < 0.001 when compared with WT rIP3R1 cell line and ###, *p* < 0.001 when compared with HEK-3KO cell line; unpaired *t* test was performed in *D* and one-way ANOVA with Tukey's test was performed in *F* (*F*_9,1568_ = 437.7, *p* < 0.0001) and *G* (*F*_9,27_ = 61.27, *p* < 0.0001).

**Figure 9:**
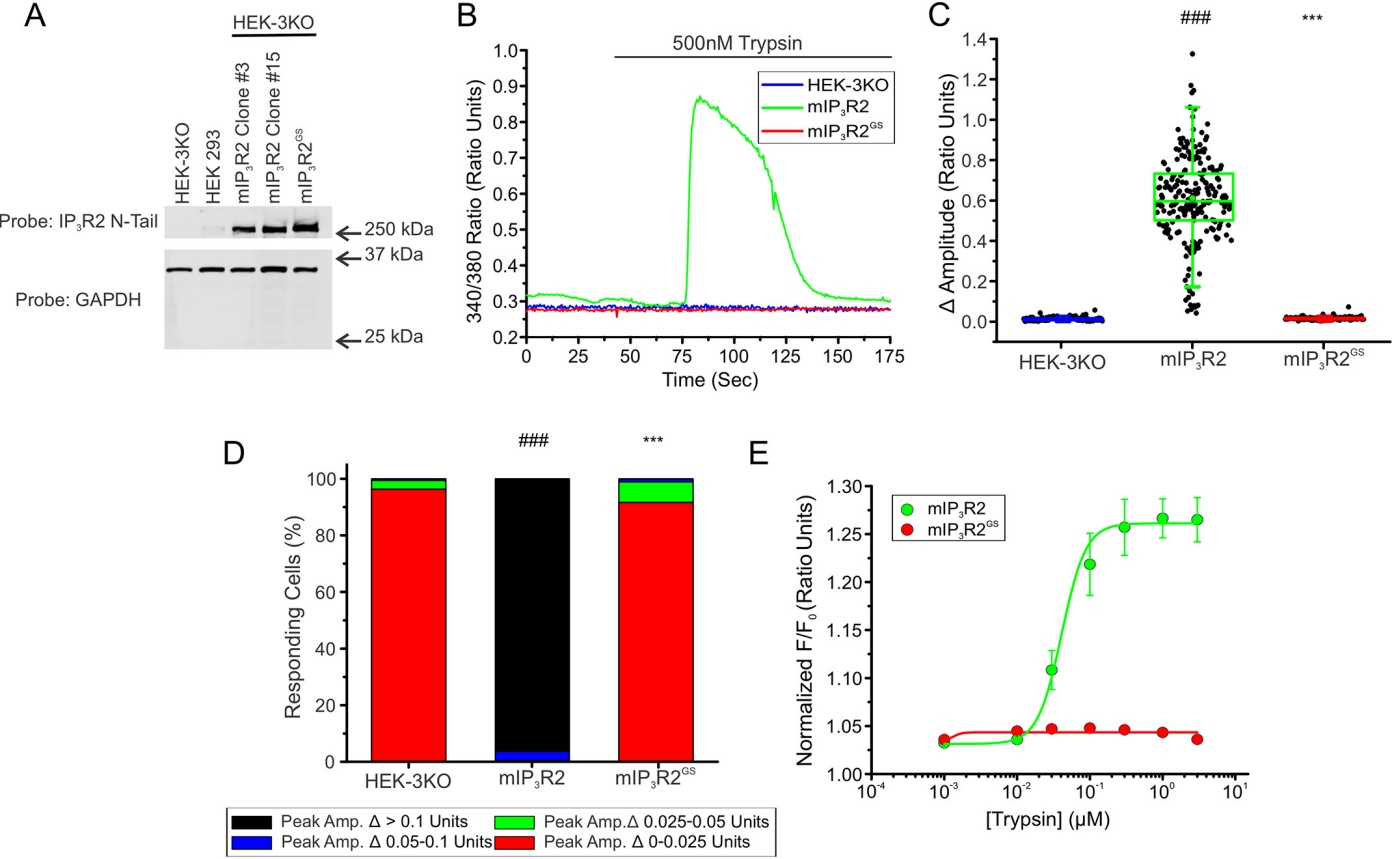
**mIP_3_R2^GS^ is predominantly nonfunctional when expressed in HEK-3KO.***A*, multiple WT mIP_3_R2 and mutant mIP_3_R2^GS^ cell lines were generated in the IP_3_R-null HEK-3KO cells and Western blotted. *B,* representative traces show Ca^2+^ signals of IP_3_R-null HEK-3KO cells (*blue*), WT mIP_3_R2 (*green*), and mIP_3_R2^GS^ (*red*) in response to trypsin (500 nm) when loaded with Fura-2/AM. *C,* scatter plots summarizing change in amplitude (peak ratio – basal ratio: average of initial 5 ratio points) for experiments similar to those shown in *B* when treated with 500 nm trypsin. *Boxes* represent the 25th, 50th, and 75th percentiles, whereas whiskers represent 5th and 95th percentiles and mean is represented by *colored circles*. *D,* stacked bar graph summarizing the percentage of amplitudes from *C,* which fall into pre-determined ranges such that only those cells with an amplitude change greater than 0.1 ratio units (*black* portion of bars) are considered to be responding to the trypsin stimulus shown in *B*. *E,* dose-response curve showing Ca^2+^ response of Fura-2/AM-loaded WT mIP_3_R2 and mIP_3_R2^GS^ cells when treated with increasing concentrations (1 nm, 10 nm, 30 nm, 100 nm, 300 nm, 1 μm, and 3 μm) of trypsin using a Flexstation3 96-well–plate reader. Data are mean ± S.E. of three (*n* = 3) independent experiments. ***, *p* < 0.001 when compared with WT rIP_3_R1 cell line and ###, *p* < 0.001 when compared with HEK-3KO cell line; one-way ANOVA with Tukey's test was performed in *C* (*F*_6,809_ = 707.1, *p* < 0.0001) and *D* (*F*_6,17_ = 335.6, *p* < 0.0001). Unless otherwise stated, all data above comes from at least *n* = 3 experiments.

We next investigated a missense mutation (G2498S) in IP_3_R2, identified in a family with generalized anhidrosis (55). IP_3_R2 is the predominant IP_3_R isoform expressed in eccrine sweat glands. Homozygous individuals were reported to have morphologically normal eccrine sweat glands but suffer from severe hyperthermia resulting from an absence of perspiration in response to physiological or pharmacological stimuli (Table S1). Notably, however, heterozygous siblings did not exhibit any symptoms of disease and thus this mutation represents a rare example of autosomal recessive inheritance of disease symptoms associated with IP_3_R mutations. The Gly-2498 residue in IP_3_R2 is located in a motif (GGG*X*G) conserved in all IP_3_R subtypes (Fig. 8*A*) as well as the superfamily of cation channels and forms the selectivity filter of the ion conducting pathway (Fig. 8*B*). This ion permeability pathway is a hot spot of other disease-associated mutations in multiple IP_3_R types (Table S4). This mutation (G2498S; designated mIP_3_R2^GS^) substitutes an evolutionarily conserved, nonpolar, hydrophobic, glycine residue, with a polar, hydrophillic serine residue and thus might reasonably be expected to disrupt the pore of the channel. Consistent with previous studies, when stably expressed in DT40-3KO cells (Fig. 8*C*), IP_3_ binding to mIP_3_R2^GS^ was comparable with WT mIP_3_R2 (Fig. 8*D*). Nevertheless, trypsin stimulation failed to elicit any Ca^2+^ release in these cells (Fig. 8, *E–G*). Again, stable expression of mIP_3_R2^GS^ in HEK-3KO cells did not alter the apparent store content of the ER (Fig. S2D) and resulted in an obvious reticular pattern of expression (Fig. S4B). However, notably, in contrast to the previous mutations investigated, when expressed in HEK-3KO cells (Fig. 9*A*) mIP_3_R2^GS^ was unable to support any measurable Ca^2+^ release (Fig. 9, *B*–*E*). Similar results were seen in other clonal lines expressing the G2498S mutations (data not shown), as well as HEK-3KO cells stably expressing a mutation in hIP_3_R1 G2506R (Fig. S6, A–F), corresponding to the neighboring glycine in the GGG*X*G motif. This mutation is associated with spinocerebellar ataxia and Gillespie syndrome. Again, differences in expression in HEK-3KO cells *versus* DT40-3KO cells may in part explain these results (Fig. S3C). Overall, these data confirm that homozygous expression of mIP_3_R2^GS^ results in a complete abrogation of IP_3_R activity and is consistent with the severe phenotype in individuals homozygous for the mutation.

**Figure 10:**
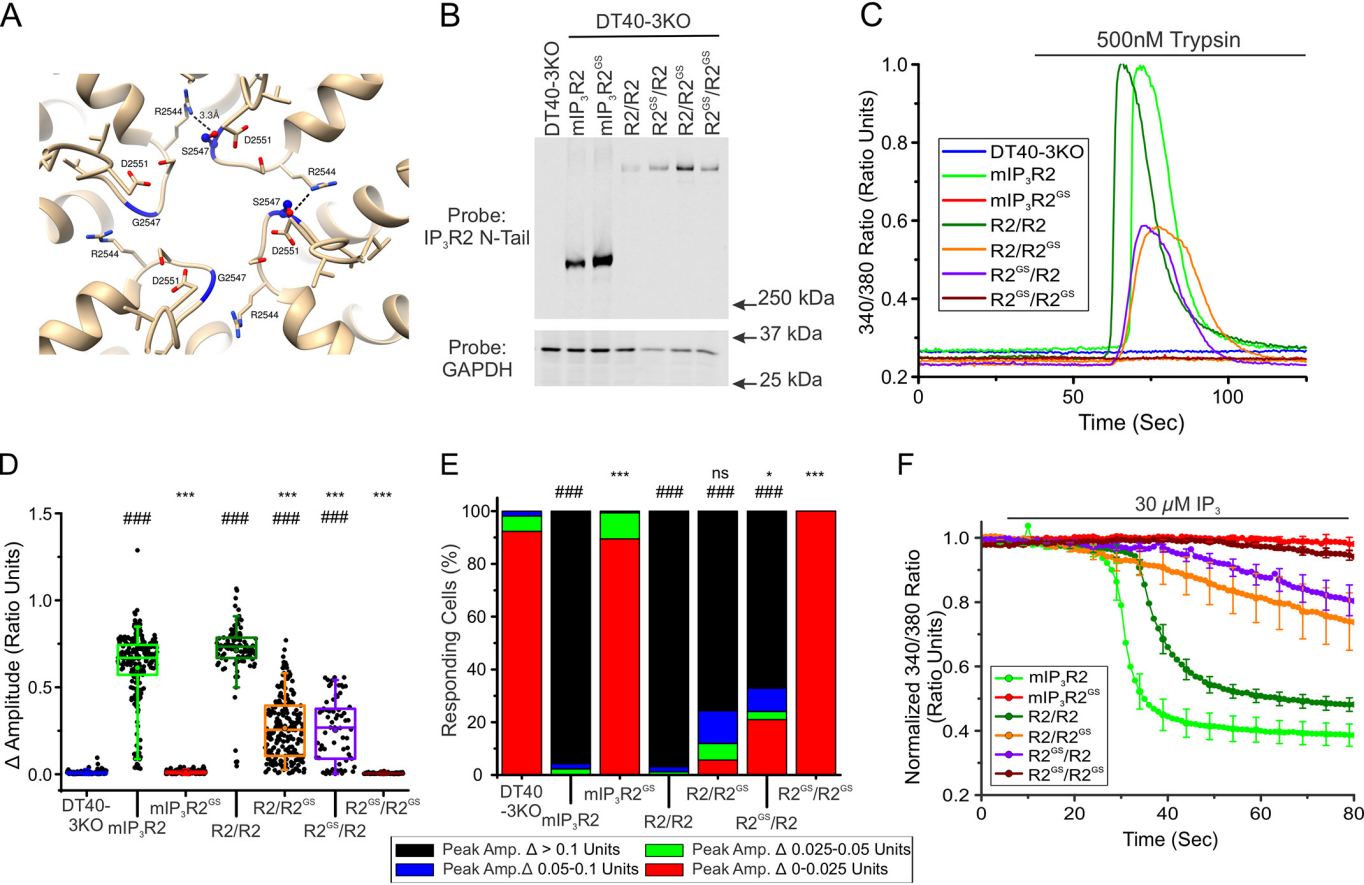
**Heterotetramers of WT mIP_3_R2 and mIP_3_R2^GS^ retain partial functionality when expressed in DT40-3KO.***A,* the current IP_3_R structural model based on the cryo-EM structure of IP_3_R1 (PDB codes 6MU1 and 6MU2) indicates that the G2498S substitution may form H bonds with neighboring Arg-2544. *B,* monomeric WT mIP_3_R2 and mutant mIP_3_R2^GS^ cell lines, as well as dimeric R2/R2, R2/R2^GS^, R2^GS^/R2, and R2^GS^/R2^GS^ cell lines generated in the IP_3_R-null DT40-3KO cells were Western blotted. *C,* representative traces show Ca^2+^ signals of IP_3_R-null DT40-3KO cells (*blue*), WT mIP_3_R2 (*green*), and mIP_3_R2^GS^ (*red*), R2/R2 (*dark green*), R2/R2^GS^ (*orange*), R2^GS^/R2 (*purple*), and R2^GS^/R2^GS^ (*dark red*) in response to trypsin (500 nm) when loaded with Fura-2/AM. *D,* scatter plots summarizing change in amplitude (peak ratio – basal ratio: average of initial 5 ratio points) for experiments similar to those shown in *C*. *Boxes* represent the 25th, 50th, and 75th percentiles, whereas whiskers represent the 5th and 95th percentiles and mean is represented by *colored circles*. *E,* stacked bar graph summarizing the percentage of amplitudes from *D,* which fall into pre-determined ranges such that only those cells with an amplitude change greater than 0.1 ratio units (*black* portion of bars) are considered to be responding to the trypsin stimulus shown in *C*. *F,* traces show Ca^2+^ signals of β-escin permeabilized WT mIP_3_R2 (*green*), and mIP_3_R2^GS^ (*red*), R2/R2 (*dark green*), R2/R2^GS^ (*orange*), R2^GS^/R2 (*purple*), and R2^GS^/R2^GS^ (*dark red*) cell lines in response to IP_3_ (30 μm) when loaded with Mag-Fura-2/AM. Data are mean ± S.E. of three (*n* = 3) independent experiments. Data for DT40-3KO, mIP_3_R2, and mIP_3_R2^GS^ in *D–-F* came from Fig. 8. *, *p* < 0.05 and ***, *p* < 0.001 when compared with corresponding WT cell line (WT mIP_3_R2 for monomers and R2/R2 cell line for dimers) and ###, *p* < 0.001 when compared with DT40-3KO cell line; one-way ANOVA with Tukey's test was performed in *D* (*F*_9,1568_ = 437.7, *p* < 0.0001) and *E* (*F*_9,27_ = 61.27, *p* < 0.0001). Unless otherwise stated, all data above comes from at least *n* = 3 experiments. *ns*, non-significant.

As the heterozygous siblings of the affected individuals were seemingly not markedly impacted by harboring the mutation, we next investigated the behavior of tetrameric channels assembled from WT and mIP_3_R2^GS^. Immunoprecipitation once again confirmed that mutated mIP_3_R2^GS^ monomers could interact with other monomeric subunits (Fig. S1, B and C). The current structural model based on the cryo-EM structure of IP_3_R1 (PDB codes 6MU1 and 6MU2) indicates that the Ser substitution for Gly-2547 (Gly-2498 in IP_3_R2) may form H bonds with the neighboring Arg-2544 residue (Fig. 10*A*), potentially resulting in constrained movement and restricted opening of the pore in a homotetramer harboring this mutant. Concatenated cDNA encoding either homodimeric mIP_3_R2-mIP_3_R2 (R2/R2) and mIP_3_R2^GS^-mIP_3_R2^GS^ (R2^GS^/R2^GS^) or heterodimeric IP_3_R2^GS^-IP_3_R2 (R2^GS^/R2 and R2/R2^GS^) were generated and stably expressed in DT40-3KO cells (Fig. 10*B*). As shown previously (38), trypsin stimulation of cells expressing both mIP_3_R2 and R2/R2 elicited vigorous Ca^2+^ release (Fig. 10*C*). Although cells expressing R2^GS^/R2^GS^ were refractory to stimulation, surprisingly, stimulation of cells expressing R2^GS^/R2 or R2/R2^GS^ exhibited robust Ca^2+^ release (Fig. 10*C* and pooled data in Fig. 10, *D* and *F*). This partial channel functionality was confirmed through stimulation of additional cell lines expressing R2^GS^/R2 or R2/R2^GS^ (data not shown). Similarly, exposure to IP_3_ in permeabilized cells also evoked robust Ca^2+^ release in WT homotetramers, intermediate Ca^2+^ release in heterotetramers, and no Ca^2+^ release in mutant homotetramers (Fig. 10*F*). These studies therefore reveal that heterotetrameric channels composed of two WT and two G2498S mutant subunits retain significant function as IP_3_-sensitive channels. Our data suggest that substitution of two or fewer Gly-Ser results in less restriction of pore movement required for gating, such that significant Ca^2+^ flux can occur that likely supports activity sufficient to render heterozygous individuals asymptomatic.

## Discussion

Mutations have been identified throughout human IP_3_R genes and are associated with numerous diseases (58, 59). How these mutations affect channel function and contribute to the pathogenesis of the disease remain, in most cases, largely unexplored. Here we showed that three different mutations in three distinct domains of the IP_3_R result in decreased channel function. The extent and penetrance of disease varies with these mutations, and our data suggest that this depends on the mechanism of channel disruption and upon the pattern of inheritance. Both factors combine to alter the stoichiometry of functional subunits in the IP_3_R tetramer and ultimately the extent of Ca^2+^ release supported by the assembled channel.

Expression of each mutation in DT40-3KO cells resulted in nonfunctional IP_3_R Ca^2+^ channels, consistent with previous studies (22, 87). In each case, the complete loss in function was not due to a difference in protein localization and expression or ER store Ca^2+^ content. These data indicate that it is unlikely that these mutations result in mislocalization, altered degradation, or a constitutively leaky channel pore. Instead, the altered functionality occurred by mutation specific mechanisms. Patients harboring a mutation of Arg-269 to both a larger tryptophan and smaller glycine present with early-onset, nonprogressive SCA. Our data show that the R269W mutation resulted in a severe disruption of IP_3_ binding. Mechanistically this is consistent with the substitution of the positively charged arginine residue with the neutral tryptophan residue impeding the coordination of negatively charged phosphate moieties of IP_3_ in the binding pocket. In addition, it is likely that the bulky rings of the tryptophan residue may also sterically hinder IP_3_ binding to other critical residues in the binding pocket core. Both factors likely severely compromise the conformational change in the binding pocket necessary for communication of the gating information required for channel opening.

The N602D mutant, in contrast to the R269W mutant, did not alter IP_3_ binding. These results correlate with data showing that this region of the receptor is outside of the IP_3_-binding core (22). This mutation, together with the T594I mutation is located between two α-helices in the ARM1 domain, adjacent to the LBD. Although it is possible that these mutations result in the alteration of IP_3_R activity by channel modulators, it is more likely that these mutations result in an alteration of the native IP_3_R conformation, which abrogates the communication of information between the binding core and the adjacent ARM1 domain. Thus, these mutations are likely best termed “gating mutants.”

Binding of IP_3_ was also not altered by mutation of G2498S. This residue is integral to the selectivity filter in the conduction pathway of the channel pore. It is likely that the mutation from a small glycine residue to a larger serine residue results in a “dead pore.” Consistent with this observation, mutation of a neighboring selectivity filter residue in IP_3_R1, Gly-2506, to both arginine (Fig. S6), and alanine also produces a similarly nonfunctional, pore mutant (26).

In contrast to expression of the mutant constructs in DT40-3KO, stable expression the R269W and N602D mutations in HEK-3KO cells resulted in channels with varying degrees of limited functionality in terms of the ability of Gα_q_-coupled agonists to elicit Ca^2+^ release in a subset of cells. Increased channel activity of these mutants observed in the HEK-3KO cells could be due in part to increased IP_3_R expression in HEK-3KO cells compared with DT40-3KO cells (Fig. S3). Conceivably, this higher expression level could facilitate receptor clustering, which would be expected to amplify small, unitary Ca^2+^ signals. Additionally, it is possible that HEK cells express so-called “licensing” factors, which are reported to render refractory IP_3_R, activatable, thus revealing a degree of functionality to mutant IP_3_R modulated in this fashion (63). Other factors could also contribute to increased functionality in HEK-3KOs including differences in accessory proteins, regulation, and the intracellular environment present in avian *versus* mammalian cells. In contrast to R269W and N602D rIP_3_R1, stable expression of G2498S mIP_3_R2 or G2506R hIP_3_R1 in HEK-3KO cells failed to yield functional channels, reflecting the severe disruption of the channel pore.

Experiments documenting the activity in both DT40-3KO and HEK-3KO of the mutants stably expressed as homotetramers provides some insight into the mechanisms underlying the altered activity. However, both the R269W and N602D are inherited in an autosomal dominant manner, as are the vast majority of documented diseases associated with IP_3_R mutations (Tables S2–S4). Given that IP_3_Rs are well documented to form heterotetramers and this is the dominant IP_3_R form when multiple species are expressed (64), it would be predicted that these patients express a variety of tetramers comprised of different ratios of WT IP_3_R1 and mutant monomers. Indeed, our data confirms that heterotetramers of WT and WT channels are readily assembled. How incorporation of mutant monomers with altered activity influences the function of the assembled channel is therefore an important question. We address this issue using concatenated IP_3_R constructs as a powerful tool to precisely control the stoichiometry of WT *versus* mutant subunits in the tetramer.

For example, heterotetramers consisting of two WT subunits and two R269W subunits are predominantly nonfunctional. Our previous studies have indicated that because of the requirement of each monomer to be liganded by IP_3_, binding deficient mutants effectively act as dominant-negative modulators if incorporated into the heterotetramer (57). These results are also consistent with previous work from our laboratory showing other IP_3_R1 mutations inherited in an autosomal dominant manner that result in Gillespie syndrome are dominant-negative (65). Moreover, these results suggest that in patients with heterozygous expression of these mutant proteins, only WT homotetramers will be able to contribute a significant amount of Ca^2+^ release. An extension of this idea, if it is assumed that tetramers form without bias, is that given a normal distribution of mutant and WT subunits in the tetramer, only a small percentage (∼9%) would be predicted to be homotetramers of WT subunits, likely severely attenuating Ca^2+^ release of the compliment of IP_3_R1 expressed.

Tetramers assembled from dimers consisting of a WT and a N602D mutant, thus expressing two mutant and two WT sub-units, respectively, were similarly largely nonfunctional. Notably, however, expression of a concatenated tetramer with three WT subunits and one mutated N602D subunit resulted in a significant increase in channel function compared with cells expressing equal numbers of mutant and WT subtypes, indicating that the N602D subunit does not function as a true “dominant-negative.” Although the channel activity of this mutant tetramer is still significantly decreased compared with tetramers with four WT IP_3_R1 subunits (WT homotetramers), these data would predict that heterozygous individuals would express a greater percentage of IP_3_R tetramers, which retain function when compared with individuals expressing the R269W mutation.

In contrast to the R269W and N602D mutants, anhidrosis caused by the G2498S mutation is one of the few documented IP_3_R disorders that is inherited in an autosomal recessive manner (55). Although individuals homozygous for the mutation exhibit a profound heat intolerance through the inability to sweat, those heterozygous for the mutation are asymptomatic. Symptomatic individuals are therefore homozygous for the expression of the mutant proteins, whereas heterozygous individuals would be predicted to express a combination of mutant and WT subunits. Our data using concatenated dimers of mutant and WT subunits provides insight into why heterozygous individuals are asymptomatic. We demonstrate that heterotetramers comprised of two WT IP_3_R2 subunits and two G2498S mutant subunits retain a significant amount of function, such that Gα_q_-coupled stimulation results in Ca^2+^ release activity intermediate between that of the WT homotetramers and the G2498S mutant homotetramers. Thus, these data indicate that in individuals heterozygous for the mutation, potentially a larger fraction of IP_3_R2 channels are functional and thus presumably provide a sufficient Ca^2+^ signal to sustain sweat secretion. Notably, numerous cell types in the periphery, including hepatocytes and exocrine cells, express IP_3_R2, usually in combination with other isoforms. These data likely also explain the highly specific phenotype observed with this mutation; whereas human sweat glands almost exclusively express IP_3_R2, there appeared to be no obvious consequences of expression of the mutant in other tissues likely because the G2498S mutation does not act in a dominant-negative fashion. Thus, heterotetramers of mutant and other WT IP_3_R subtypes would not be predicted to be substantially disabled and thus impact cellular function. In total, studies presented here using concatenated subunits provide unprecedented insight into how mutant IP_3_R may function in heterozygous individuals and help demystify these challenging channelopathies.

## Experimental procedures

### Reagents

Antibodies generated by Pocono Rabbit Farms and Laboratories included rabbit polyclonal antibodies for IP_3_R2 against amino acids 320-338 (NT2) and amino acids 2686-2701 in mouse IP_3_R2 (CT2), as well as a rabbit polyclonal antibody against the C-terminal 19 aa of IP_3_R1 (CT1) (66). Reagents used for SDS-PAGE were obtained from Bio-Rad. Protein A/G-agarose beads were from Santa Cruz. Alexa Fluor 488 and Dylight™ 800CW secondary antibodies were from Thermo Scientific. Restriction enzymes and DNA T4 ligase were purchased from New England Biolabs. β-Mercaptoethanol, chicken serum, penicillin/streptomycin, RPMI 1640 media, and G418 sulfate were obtained from Life Technologies. Fetal bovine serum and goat serum were from Gibco. Fura2-AM was from TEFLABS. All other chemicals were obtained from Sigma unless otherwise indicated.

### Plasmid construction

Mutagenesis and all DNA modifications were carried out using *Pfu* Ultra II Hotstart 2× Master Mix (Agilent). Primers used in this study were synthesized by Integrated DNA Technologies (IDT) (Table S5). A QuikChange mutagenesis protocol was used to introduce R269W in cDNAs encoding the rat IP_3_R1 (rIP_3_R1; NM_001270596) in pDNA3.1 using mutagenic primers (primers 1 and 2). These primers encode R269W, in addition to silently introducing the indicated restriction site (Table S5). Similarly, two mutagenesis primers were used to introduce N602D into rIP_3_R1 (primers 5 and 6), whereas mutagenesis primers were used to introduce R269W (primers 3 and 4), T494I (primers 7 and 8), and G2506R (primers 9 and 10 for G > A and primers 11 and 12 for G > C) into human IP_3_R1 (hIP_3_R1; NM_001099952) in pCDNA3.1 (obtained from Annetta Wronska at Columbia University). Finally, primers 13 and 14 introduced G2498S in mouse IP_3_R2 (mIP_3_R2; NM_019923) in pcDNA3.1. The coding regions for all constructs were confirmed by sequencing.

IP_3_R1 and IP_3_R2 concatenated constructs were generated as previously described in detail (38, 64, 67). Briefly, the cDNAs of the corresponding IP_3_R subunits, designated as head and tail, in pcDNA3.1 vector was modified and subcloned in a tandem fashion, between the two arms of the pJAZZ mamm linear vector (Lucigen). The resultant construct encodes one ORF consisting of two IP_3_R subunits connected with a 14-amino acid linker. Similarly, tetrameric IP_3_R1 constructs were generated using four modified IP_3_R1 subunits (numbered I-IV). The resulting cDNA forms one reading frame encoding four IP_3_R1 subunits with 14 amino acid linkers separating the subunits. To create concatenated constructs harboring one or more mutant subunits, the corresponding mutations were introduced in the individual subunits before assembling the concatenated constructs.

### Cell culture and transfection

DT40-3KO, chicken B lymphocyte cells engineered through homologous recombination for the deletion of the three endogenous IP_3_R isoforms (56), were grown at 39 °C with 5% CO_2_ in RPMI 1640 media supplemented with 1% chicken serum, 10% fetal bovine serum, 100 units/ml of penicillin, 100 µg/ml of streptomycin. DT40-3KO transfection was performed as described before (66). In brief, 5 million cells were washed once with PBS and electroporated with 5-10 µg of DNA using Amaxa cell nucleofector kit T (Lonza laboratories). Cells were allowed to recover for 24 h at 39˚C with 5% CO_2_, and then passed into 96-well–plates containing media supplemented with 2 mg/ml of G418. Clones expressing the desired constructs were identified by immunoblots 10-14 days after transfection. HEK-3KO, HEK293 cells engineered in our laboratory though CRISPR/Cas9 for the deletion of the three endogenous IP_3_R isoforms were grown at 37 °C with 5% CO_2_ in Dulbecco's modified Eagle's medium supplemented with 10% fetal bovine serum, 100 units/ml of penicillin, 100 µg/ml of streptomycin.

HEK-3KO transfection was also performed similarly to as previously described (57). In brief, 1 million cells were washed once with PBS and electroporated with 5-10 µg of DNA using Amaxa cell nucleofector kit T (Lonza laboratories). Cells recovered for 48 h at 37 °C with 5% CO_2_, and then were passed into new 10-cm plates containing media supplemented with 2 mg/ml of G418. After 7 days of selection, immunoblot was performed to assess expression of the desired constructs in these stable HEK cell pools. Following confirmation of expression, cells were either picked and transferred to new 24-well– plates or diluted at various concentrations into 96-well–plates or containing media supplemented with 2 mg/ml of G418. Monoclonal lines were identified 7 days after dilution and clones expressing the desired constructs were identified by immunoblots 10-14 days after identification.

### Cell lysis, co-immunoprecipitation, and SDS-PAGE analyses

Following treatment, cells were harvested by centrifugation, washed once with PBS, and solubilized in cell lysis buffer containing 10 mm Tris-HCl, 10 mm NaCl, 1 mm EGTA, 1 mm EDTA, 1 mm NaF, 20 mm Na_4_P_2_O_7_, 2 mm Na_3_ VO_4_, 1% Triton X-100 (v/v), 0.5% sodium deoxycholate (w/v), and 10% glycerol supplemented with protease inhibitors. Lysates were incubated for 30 min on ice and cleared by centrifugation at 16,000 × *g* for 10 min at 4 °C. Protein concentrations in cleared lysates were determined using Dc protein assay kit (Bio-Rad). Cleared supernatants were rocked with the indicated antibodies and 30 μl of protein A/G slurry overnight. Immunocomplexes were washed five times and resuspended in gel loading buffer. Proteins were fractionated on SDS-PAGE gel and transferred to a nitrocellulose membrane, which was probed with the indicated primary antibodies and corresponding secondary antibodies. Membranes were imaged with an Odyssey IR imaging system (LICOR Biosciences).

### Immunocytochemistry and confocal microscopy

Stable HEK cell lines were plated on poly-d-lysine–coated coverslips. When roughly 50% confluent cells were fixed using ice-cold methanol at −20 °C or paraformaldehyde at room temperature for 10 min. Subsequently, coverslips were washed with PBS and cells were blocked in goat serum or BSA for 1.5 h. Following blocking, cells were incubated in primary antibody (IP_3_R1 or IP_3_R2) and DAPI overnight at 4 °C. The following day, primary antibody was removed, and coverslips were washed 3 times with PBS for 10 min with gentle rocking. Subsequently, the secondary antibody conjugated to Alexa Fluor 488 or 594 was incubated for 1 h at room temperature with gentle rocking. After incubation, coverslips were washed with PBS and mounted on slides. After allowing slides to dry, coverslips were sealed onto slides and imaged using confocal microscopy.

### Measurement of cytosolic Ca^2+^ in intact cells

Single cell Ca^2+^ imaging was performed as described previously (66). DT40-3KO and HEK-3KO cells expressing IP_3_R constructs were washed once with imaging buffer (10 mm HEPES, 1.26 mm Ca^2+^, 137 mm NaCl, 4.7 mm KCl, 5.5 mm glucose, 1 mm Na_2_HPO_4_, 0.56 mm MgCl_2_, pH 7.4). Cells were then resuspended in imaging buffer containing 2 μm Fura2-AM and placed on a coverslip for 20 min at room temperature to allow for fluorescent dye loading and attachment of cells to the glass coverslip. Cells were then perfused with imaging buffer and stimulated with the desired agonist. Ca^2+^ imaging was performed using an inverted epifluorescence Nikon microscope with a ×40 oil immersion objective. Cells were alternately excited at 340 and 380 nm, and emission was monitored at 505 nm. Images were captured every second with an exposure of 10 ms and 4 × 4 binning using a digital camera driven by TILL Photonics software. Image acquisition was performed using TILLvisION software and data were exported to Microsoft excel. Experiments were repeated at least three times.

Population-based Ca^2+^ imaging was performed in intact HEK cells by loading cells with 4 μm Fura-2 AM in culture medium. After 1 h, cells were harvested and subsequently washed, resuspended in imaging buffer, and dispensed into a black-walled flat-bottom 96-well–plate (∼300,000 cells/well). The plate was spun at 200 × *g* for 2 min to plate cells to the bottom of each well. The plate rested for 30 min prior to commencing the assay. Fluorescence imaging was carried out using FlexStation 3 from Molecular Devices (excitation alternated between 340 and 380 nm and emission 510 nm) and analyzed by using SoftMax© Pro Microplate Data Acquisition and Analysis software to determine peak of the response. Data were averaged from at least 3 individual plates and curves fitting was done using a logistic dose-response equation in the OriginPro 6.1 software.

### Single cell-permeabilized DT40 cell IP_3_-induced Ca^2+^ release (IICR) assays

DT40-3KO cells stably expressing defined IP_3_R constructs were loaded with 20 μm MgFura-2 AM for 50–60 min and subsequently permeabilized by superfusion of 40 μm β-escin in imaging buffer as previously described (57, 64). The duration of permeabilization depended on the flow rate, and care was taken to prevent excessive (internal membrane) permeabilization by careful monitoring of the fluorescence. Permeabilized cells were then washed in ICM without β-escin for 15 min to facilitate removal of cytosolic dye. The internal stores of permeabilized cells were loaded by activating SERCA through superfusion with ICM containing 1.4 mm MgCl_2_, 3 mm NaATP, and 0.65 mm CaCl_2_ (free [Ca^2+^] of 200 nm (MaxChelator freeware)). Upon stabilization of fluorescence, MgCl_2_ was removed from the superfused solution to disable SERCA. Varying concentrated IP_3_ were applied through superfusion to induce unidirectional Ca^2+^ release from internals stores. After washing out IP_3_, stores were repeatedly refilled and released to allow for repeated stimulations. Each experiment contained between 30 and 60 cells in a field of view and performed a minimum of three times. The initial rates of Ca^2+^ release were determined by fitting the release curves of each individual cell to a single exponential function (OriginPro 6.1). Concentration-response curves were plotted using the determined rates. All imaging was performed on an inverted epifluorescence Nikon microscope with a ×40 oil immersion objective. Cells were alternately excited at 340 and 380 nm, and emission was monitored at 505 nm. Images were captured every 5 s during permeabilization, loading, and disabling and every second during release. This was done with an exposure of 10 ms and 4 × 4 binning using a digital camera driven by TILL Photonics software.

### Competitive IP_3_-binding assay

DT40-3KO cells stably expressing the indicated IP_3_R constructs were washed once with PBS and lysed in Igepal lysis buffer containing (120 mm NaCl, 50 mm Tris-HCl, 0.5% Igepal (v/v), 1 mm EDTA) supplemented with a mixture of protease inhibitors. Lysates were incubated on ice for 30 min and cleared by centrifugation at 16,000 × *g* for 10 min at 4 °C. IP_3_R proteins were immunoprecipitated overnight using anti-IP_3_R and protein A/G PLUS-agarose beads. Immunocomplexes were washed three times with lysis buffer and twice with binding buffer (50 mm Tris-base, 1 mm EDTA, pH 8, plus 1 mm β-mercaptoethanol) and equally distributed into 1.5-ml microcentrifuge tubes. One pair of tubes was immunoblotted using SDS-PAGE to ensure equal immunoprecipitation of WT and mutant protein, whereas all others underwent the binding reaction consisting of a 100-μl volume containing equal amounts of immunoprecipitated proteins, 2.5 nm tritiated IP_3_ ([^3^H]IP_3_) in the presence of different concentrations of unlabeled IP_3_. Tubes were incubated for 1 h at 4 °C with mixing every 10 min. Finally, tubes were centrifuged at 16,000 × *g*, supernatants were removed, and 500 µl of 1% of SDS was added to each tube. The contents of the tubes were transferred to vials containing scintillation liquid after 12 h and bound radioactivity were measured using liquid scintillation counter. Nonspecific binding was calculated as the amount of bound radioactivity in the presence of 50 μm unlabeled IP_3_. Specific binding is determined by subtracting nonspecific binding from the CPM values obtained in other conditions. Total specific binding was determined as the binding observed in the absence of unlabeled IP_3_. Normalized specific binding from 3 experiments were averaged and curves were fit using a logistic dose-response equation using the OriginPro 6.1 software.

### Data analysis and statistical analysis

Data obtained were exported into Microsoft Excel where appropriate ratios, normalizations, and averages were calculated. Statistical analysis was performed in GraphPad Prism for experiments comparing two cell lines (unpaired *t* test) and three or more cell lines (one-way ANOVA with Tukey's test). Logistic fits, as well as IC_50_ were calculated using OriginPro 6.1.

## Data availability

All constructs used are available freely on request. All data needed to evaluate the conclusions are presented in the paper or Supporting material.

## Footnotes

1 The nucleotide sequences, and thus amino acid numbering, used throughout this paper are: rIP_3_R1, NM_001007235; hIP_3_R1, NM_001099952; mIP_3_R2, NM_019923. See Table S6 for alignment guide of these sequences with other commonly used sequences.
