# Supplementary material for: Disease-associated mutations in inositol 1,4,5-trisphosphate receptor subunits impair channel function
Source: J Biol Chem. 2021 Jan 13;295(52):18160–78. doi: 10.1074/jbc.RA120.015683 (PMC7939385; doi:10.1074/jbc.RA120.015683)
Supplement: Supplementary file 1 [file mmc1.pdf]

Impaired function of inositol 1,4,5-trisphosphate receptor channels harboring disease-associated mutant subunits

Lara E. Terry, Kamil J. Alzayady, Sundeep Malik and David I. Yule

Supplementary Materials

Experimental Procedures

Measurement of ER Store Ca^{2+} content in Intact Cells

Table S1. Disease phenotypes of discussed IP_3R mutations in ligand binding domain, regulatory and coupling domain, and channel domain
Table S2. Molecular characteristics of disease associated IP_3R mutations in the ligand binding domain
Table S3. Molecular characteristics of disease associated IP_3R mutations in the regulatory and coupling domain
Table S4. Molecular characteristics of disease-associated IP_3R mutations in the c-terminal channel domain
Table S5. Mutagenesis primers
Table S6. Corresponding residues in the other sequences

Figure S1. Mutant IP_3R immunoprecipitated with wild-type IP_3R
Figure S2. Stable expression of IP_3R mutants tested do not alter ER Ca^{2+} store content
Figure S3. Mutant expression is increased in HEK-3KO cells compared to DT40-3KO cells
Figure S4. rIP_3R1^{ND} and mIP_3R2^{GS} constructs localize to the ER membrane when stably expressed in HEK-3KO cells
Figure S5. rIP_3R1^{TI} is non-functional when expressed in HEK-3KO cells
Figure S6. rIP_3R1^{GR} is non-functional when expressed in HEK-3KO cells

Supplementary Materials:

Experimental Procedures

Measurement of ER Store Ca^{2+} content in Intact Cells

HEK cells were initially prepared as indicated above for population-based imaging of cytoplasmic Ca^{2+}; however, prior to dispensing of cells in 96-well plate (~ 300,000 cells/well), cells were washed with and plated in a 0.1 mM Ca^{2+} imaging buffer. The plate was centrifuged at 200g for 2 minutes to plate cells to the bottom of each well. The plate rested for 30 min prior to commencing the assay. Fluorescence imaging was carried out using FlexStation 3 from Molecular Devices (excitation alternated between 340 nm and 380 nm and emission 510 nm). To force an extracellular Ca^{2+} concentration of 200nM, 0.1365 mM EGTA was added to the wells (calculated using MaxChelator). Subsequently, 30 µM CPA was added to block SERCA and allow for emptying of the ER Ca^{2+} store. Results were exported to Excel where ratio of 340/380 values and area under the curve were calculated. Data was averaged from 3 experimental runs.

<table>
<tr><th>Mutation[a]</th><th colspan="2">Original Residue (Base Change) & Reference Sequence[b]</th><th>Isoform</th><th>Domain</th><th>Conserved[c]</th></tr>
<tr><td rowspan="3">R269G</td><td colspan="2">R269G (c.805C>G) in NM_001099952.2</td><td>Itpr1</td><td>Ligand Binding Domain</td><td>Yes</td></tr>
<tr><th>Diagnosis</th><th>Individuals Affected</th><th colspan="2">Reported Phenotype</th><th>Reference</th></tr>
<tr><td>SCA29</td><td>1 de novo case[d]</td><td colspan="2">Infantile-onset non-progressive ataxia, hypotonia, gross motor delay and mild cognitive impairment</td><td>Zambonin et al. 2017</td></tr>
<tr><th>Mutation</th><th colspan="2">Original Residue (Base Change) & Reference Sequence</th><th>Isoform</th><th>Domain</th><th>Conserved</th></tr>
<tr><td rowspan="6">R269W</td><td colspan="2">R269W (c.805C>T) in NM_001168272.1& NM_001099952.2</td><td>Itpr1</td><td>Ligand Binding Domain</td><td>Yes</td></tr>
<tr><th>Diagnosis</th><th>Individuals Affected</th><th colspan="2">Reported Phenotype</th><th>Reference</th></tr>
<tr><td>Autosomal dominant NPCA</td><td>Mother & 2 Sons (inherited)</td><td colspan="2">Infantile-onset cerebellar ataxia, cerebellar atrophy, delayed motor development, hypotonia, nystagmus, postural tremor, and slurred speech. Intellectual disability in one son.</td><td>Barresi et al. 2016</td></tr>
<tr><td>SCA29</td><td>1 de novo case[d]</td><td colspan="2">Infantile-onset non-progressive ataxia, hypotonia, gross motor delay and mild cognitive impairment</td><td>Zambonin et al. 2017</td></tr>
<tr><td>Ataxic Cerebral Palsy</td><td>Mother & Daughter (inherited)</td><td colspan="2">Infantile-onset cerebellar ataxia, intellectual disability, delayed motor development, cerebellar atrophy, myoclonic jerks, myokymia, postural tremor, hypotonia, and dysarthria</td><td>Das et al. 2017</td></tr>
<tr><td>EOA</td><td>2 de novo cases</td><td colspan="2"><u>Case 1 (P2):</u> Non-progressive infantile onset ataxia, delayed motor development, mild cerebellar atrophy (vermis), isolated dyscalculia, slowing of horizontal saccades (COMA), convergent strabismus, and myoclonus in the extremities & trunk
<u>Case 2 (P6):</u> Non-progressive infantile onset ataxia, delayed motor development, nystagmus, hypotonia, mild cerebellar atrophy, cognitive impairment, & strabismus</td><td>Synofzik et al. 2018</td></tr>
<tr><th>Mutation</th><th colspan="2">Original Residue (Base Change) & Reference Sequence</th><th>Isoform</th><th>Domain</th><th>Conserved</th></tr>
<tr><td rowspan="3">T594I</td><td colspan="2">T594I (c.1781C>T) in NM_001099952.2</td><td>Itpr1</td><td>Regulatory and Coupling Domain</td><td>Yes</td></tr>
<tr><th>Diagnosis</th><th>Individuals Affected</th><th colspan="2">Reported Phenotype</th><th>Reference</th></tr>
<tr><td>Sporadic infantile-onset SCA</td><td>1 Sporadic Case</td><td colspan="2">Motor developmental delays, moderate cognitive deficits, ataxia, oculomotor apraxia, vertical and horizontal nystagmus observed as early as the first day of life, general hypotonia and postural tremor of the head, arms, and trunk, and diffuse cerebellar atrophy, as well as atrophy of the pontine tegmentum. No progressive motor or intellectual deterioration was observed during follow-up</td><td>Sasaki et al. 2015</td></tr>
<tr><th>Mutation</th><th colspan="2">Original Residue (Base Change) & Reference Sequence</th><th>Isoform</th><th>Domain</th><th>Conserved</th></tr>
<tr><td>N602D</td><td colspan="2">N602D (c. 1804A>G) in NM_001099952.2 & (c.1759A>G) in NM_001168272.1</td><td>Itpr1</td><td>Regulatory and Coupling Domain</td><td>Yes</td></tr>
</table>

Mutation	Diagnosis	Individuals Affected	Reported Phenotype	Reference
	Autosomal Dominant CNPCA/SCA29	4 Related Individuals (inherited)	<u>Proband:</u> Non-progressive ataxia, delayed gross motor development, cerebellar atrophy, cognitive delay, gaze-evoked nystagmus, epilepsy, hypotonia, truncal titubation, and limb ataxia<br><u>Father:</u> Delayed gross motor milestones, academic difficulties, saccadic eye movements with end range nystagmus, dysarthria, gait and limb ataxia, intension tremor, and diffuse cerebellar atrophy	Huang *et al.* 2012<br>Zambonin *et al.* 2017
	Ataxic Cerebral Palsy	1 de novo case	Non-progressive ataxia, delayed gross motor development, moderate intellectual disability, hypotonia, and normal brain imaging	Parolin Schnekenberg *et al.* 2015
**Mutation**	**Original Residue (Base Change) & Reference Sequence**	**Isoform**	**Domain**	**Conserved**
G2506R	G2506R (c.7516G>A) in NM_001099952.2 & G2539R (c.7615G>A/C) in NM_001168272.1	*Itpr1*	Selectivity Filter	Yes
	**Diagnosis**	**Individuals Affected**	**Reported Phenotype**	**Reference**
	GS (c.7615G>A)	5 de novo cases	<u>P261348:</u> Delayed ability to sit and walk independently, speech delay, mild learning difficulties, ataxia, hypotonia, bilateral iris hypoplasia, foveal hypoplasia, mild visual impairment, cerebellar hypoplasia/atrophy, gall stones, and scoliosis<br><u>P2021_2021:</u> Delayed ability to sit independently, moderate speech delay, mild to moderate learning difficulties, ataxia, hypotonia, bilateral iris hypoplasia, and cerebellar hypoplasia/atrophy<br><u>P2018_2018:</u> Ataxia, hypotonia, bilateral iris hypoplasia, and cerebellar hypoplasia/atrophy not known<br><u>P5284_5284:</u> Delayed ability to sit independently, speech delay, mild learning difficulties, ataxia, hypotonia, bilateral iris hypoplasia, mild visual impairment, cerebellar hypoplasia/atrophy, patent foramen ovale, and mild pulmonary valve stenosis<br><u>P5285_5285:</u> Moderate learning difficulties, ataxia, hypotonia, bilateral iris hypoplasia, and cerebellar hypoplasia/atrophy	McEntagart *et al.* 2016
	SCA29[d]	1 de novo case<br>1 inherited case	Infantile-onset non-progressive ataxia, hypotonia, gross motor delay and mild cognitive impairment	Zambonin *et al.* 2017
	GS (c.7615G>C)	1 de novo case	Delayed ability to sit and walk independently, severe speech delay, learning difficulties, ataxia, bilateral iris hypoplasia, cerebellar hypoplasia/atrophy, and gastrointestinal reflux	McEntagart *et al.* 2016
**Mutation**	**Original Residue (Base Change) & Reference Sequence**	**Isoform**	**Domain**	**Conserved**
G2498S	G2498S (c.7492G>A) in NM_002223.2	*Itpr2*	Selectivity Filter	Yes
	**Diagnosis**	**Individuals Affected**	**Reported Phenotype**	**Reference**
	Anhidrosis	5 Homozygous cases & 5 Heterozygous cases (inherited)	<u>Homozygous Individuals:</u> Anhidrosis with severe heat intolerance leading to increased heart rate and skin and ear canal temperatures<br><u>Heterozygous Individuals:</u> Asymptomatic	Klar *et al.* 2014

Table S1. Disease phenotypes of discussed IP_3R mutations in ligand binding domain, regulatory and coupling domain, and channel domain.
Abbreviations: CNPCA: Congenital non-progressive cerebellar ataxia; EOA: early onset ataxia; GS: Gillespie Syndrome; NPCA: non-progressive congenital ataxia; SCA: Spinocerebellar Ataxia.
[a] Mutations reported in the reference sequence of NP_001093422 (IP_3R1), NP_002214 (IP_3R2), or NP_002215 (IP_3R3). If not originally reported in this reference sequence, multiple sequence alignment was utilized to find residue in appropriate reference sequence.
[b] This indicates the residue, base change, and reference sequence in which the mutation was originally discovered.
[c] This refers to whether residue is conserved among human, rat, and mouse sequences of all three IP_3R isoforms.
[d] Details of individuals phenotype beyond general SCA29 symptoms not provided.

Mutation[a]	Original Residue (Base Change) & Reference Sequence[b]	Isoform	Domain	Conserved[c]	Diagnosis	Individuals Affected	Reference
R241K	R241K (c.722G>A) in NM_001168272.1	*Itpr1*	LBD	Yes	Autosomal dominant NPCA	Mother & Daughter (inherited)	Barresi *et al.* 2016
E246K	E246K (c.736G>A) in NM_001099952.2	*Itpr1*	LBD	Yes	EOA	1 de novo case (Validation Cohort)	Synofzik *et al.* 2018
T267M	T267M (c.800C>T) in NM_001099952.2	*Itpr1*	LBD	Yes	Sporadic infantile-onset SCA	1 Sporadic Case	Ohba *et al.* 2013 Sasaki *et al.* 2015
					SCA29	2 Sporadic Cases	Zambonin *et al.* 2017
					EOA	1 de novo case (Validation Cohort)	Synofzik *et al.* 2018
T267R	T267R (c.800C>G) in NM_001099952.2	*Itpr1*	LBD	Yes	Sporadic infantile-onset SCA	1 Sporadic Case	Sasaki *et al.* 2015
R269G	R269G (c.805C>G) in NM_001099952.2	*Itpr1*	LBD	Yes	SCA29	1 de novo case	Zambonin *et al.* 2017
R269W	R269W (c.805C>T) in NM_001168272.1& NM_001099952.2	*Itpr1*	LBD	Yes	Autosomal dominant NPCA	Mother & 2 Sons (inherited)	Barresi *et al.* 2016
					SCA29	1 de novo case	Zambonin *et al.* 2017
					Ataxic Cerebral Palsy	Mother & Daughter (inherited)	Das *et al.* 2017
					EOA	2 de novo case (Validation Cohort)	Synofzik *et al.* 2018
S277I	S277I (c.830G>T) in NM_001099952.2	*Itpr1*	LBD	Yes	SCA15 (with early onset)	1 Sporadic Case	Fogel *et al.* 2014
					Sporadic infantile-onset SCA	1 Sporadic Case	Sasaki *et al.* 2015
					SCA29	1 de novo case	Zambonin *et al.* 2017
K279E	K279E (c.835A>G) in NM_001099952.2	*Itpr1*	LBD	No	SCA29	1 de novo case	Zambonin *et al.* 2017
A280D	A280D (c.839C>A) in NM_001168272.1	*Itpr1*	LBD	Yes	Autosomal dominant NPCA	1 de novo case	Barresi *et al.* 2016
Exon 14 Splice Mutation	Exon 14 (c.1207-2A-T) in *itpr1*	*Itpr1*	LBD	Yes	Autosomal dominant CNPCA	4 Related Individuals (inherited)	Wang *et al.* 2017
K417_418Ins	K417_418Ins (c.1252-1G>T) in NM_001099952.2	*Itpr1*	LBD	No	SCA29	1 de novo case	Zambonin *et al.* 2017
V494I	V494I (c.1480G>A) on NG_016144.1	*Itpr1*	LBD	Yes	SCA15	1 Individual	Ganesamoorthy *et al.* 2009
E512K	E497K (c.1889G>A) in NM_001168272.1	*Itpr1*	LBD	No	Autosomal dominant NPCA	1 de novo case	Barresi *et al.* 2016

R568G	R568G (c.1702A>G) in NM_001099952.2	*Itpr1*	LBD	Yes	EOA	1 de novo case (Also inherited M1144V & A2069S)	Synofzik *et al.* 2018

Table S2. Molecular characteristics of disease associated IP_3R mutations in the ligand binding domain. Abbreviations: CNPCA: Congenital non-progressive cerebellar ataxia; EOA: early onset ataxia; LBD: Ligand Binding Domain; NPCA: non-progressive congenital ataxia; SCA: Spinocerebellar Ataxia.

[a] Mutations reported in the reference sequence of NP_001093422 (IP_3R1), NP_002214 (IP_3R2), or NP_002215 (IP_3R3). If not originally reported in this reference sequence, multiple sequence alignment was utilized to find residue in appropriate reference sequence.

[b] This indicates the residue, base change, and reference sequence in which the mutation was originally discovered.

[c] This refers to whether residue is conserved among human, rat, and mouse sequences of all three IP_3R isoforms.

Mutation[a]	Original Residue (Base Change) & Reference Sequence[b]	Isoform	Domain	Conserved[c]	Diagnosis	Individuals Affected	Reference
T594I	T594I (c.1781C>T) in NM_001099952.2	*Itpr1*	R/C	Yes	Sporadic infantile-onset SCA	1 Sporadic Case	Sasaki *et al.* 2015
N602D	N602D (c. 1804A>G) in NM_001099952.2 & (c.1759A>G) in NM_001168272.1	*Itpr1*	R/C	Yes	Autosomal Dominant CNPCA/SCA29	4 Related Individuals (inherited)	Huang *et al.* 2012 Zambonin *et al.* 2017
					Ataxic Cerebral Palsy	1 de novo case	Parolin Schnekenberg *et al.* 2015
R728*	R728* (c.2182C>T) in NM_001099952.2	*Itpr1*	R/C	No	GS	1 de novo case	Gerber *et al.* 2016
A911V	A911V (c. 2732C>T) in NM_001099952.2	*Itpr1*	R/C	No	EOA	1 inherited case (Non-ataxic parents)	Synofzik *et al.* 2018
					Hereditary Spastic Paraplegia	7 Individuals in 2 Unrelated Families	Elert-Dobkowska *et al.* 2019
N984fs	N984fs (c.2952_2953insTATA) in NM_001099952.2	*Itpr1*	R/C	No	GS + Cardiovascular Symptoms	2 Siblings	Carvalho *et al.* 2017
P1074L	P1059L (c.8581C>T) in NM_002222.5	*Itpr1*	R/C	No	SCA15	Multiple Individuals (inherited)	Hara *et al.* 2008
M1064V	M1064V (c.3190A>G) in NM_002224.3	*Itpr3*	R/C	Yes	Neuropathy	1 de novo case	Lassuthova *et al.* 2016
M1144V	M1144V (c.3430A>G) in NM_001099952.2	*Itpr1*	R/C	No	EOA	1 inherited case (Also de novo R568G & inherited A2069S)	Synofzik *et al.* 2018
					Hereditary Spastic Paraplegia	1 case - inherited vs de novo unknown (A2069S also present)	Elert-Dobkowska *et al.* 2019
T1386M	T1386M (c.4157C>T) in NM_001099952.2	*Itpr1*	R/C	No	SCA29	1 de novo case	Zambonin *et al.* 2017
T1424M	T1424M (c.4271C>T) in NM_002224.3	*Itpr3*	R/C	Yes	Neuropathy	1 de novo case	Schabhuttl *et al.* 2014
S1493D	S1487D(c.4459_4460delinsGA) in NM_001168272.1	*Itpr1*	R/C	Yes	Ataxic Cerebral Palsy	1 de novo case	Parolin Schnekenberg *et al.* 2015
V1553M	V1553M (c.4657G>A) in NM_001099952.2	*Itpr1*	R/C	No	Autosomal Dominant CNPCA/SCA29	20 Related Individuals (inherited)	Dudding *et al.* 2004 Huang *et al.* 2012 Zambonin *et al.* 2017
					SCA29	5 Related Individuals (inherited)	Shadrina *et al.* 2016

Q1558*	Q1558* (c.4672C>T) in NM_001099952.2	*Itpr1*	R/C	No	GS	1 de novo case	Gerber *et al.* 2016
E1666D	E1666D (c.4998A>C) in NM_001099952.2	*Itpr1*	R/C	No	EOA	1 inherited case (Non-ataxic parents)	Synofzik *et al.* 2018
L1787P	L1827P (c.5360T>C) in P29994.2	*Itpr1*	R/C	Yes	SCA29 (Autosomal recessive)	6 Homozygous Cases & 5 Heterozygous Cases (inherited)	Klar *et al.* 2017
D1839N	D1839N (c.5515G>A) in NP_002214.2	*Itpr2*	R/C	No	FIHP	Mother & daughter	Cetani *et al.* 2019
E2061G	E2094G (c.6281A>G) in NM_001168272.1	*Itpr1*	R/C	Yes	GS	Mother & Daughter (inherited)	McEntagart *et al.* 2016
E2061Q	E2094Q (c.6280G>C) in NM_001168272.1	*Itpr1*	R/C	Yes	GS	1 de novo case	McEntagart *et al.* 2016
E2061K	E2094K (c.6280G>A)	*Itpr1*	R/C	Yes	GS with minor cerebellar involvement & no intellectual disability	1 de novo case	Stendel *et al.* 2019
A2069S	A2069S (c.6205G>T) in NM_001099952.2	*Itpr1*	R/C	Yes	EOA	1 inherited case (Also de novo R568G & inherited M1144V)	Synofzik *et al.* 2018
					Hereditary Spastic Paraplegia	1 case - inherited vs de novo unknown (M1144V also present)	Elert-Dobkowska *et al.* 2019
G2102Valfs5*/ A2221Valfs23*	G2102Valfs5*/ A2221Valfs23* (c.6366+3A>T/ c.6664+5G>T) in NM_001099952.2	*Itpr1*	R/C	No	GS	1 de novo case	Gerber *et al.* 2016

Table S3. Molecular characteristics of disease associated IP_3R mutations in the regulatory and coupling domain.

Abbreviations: CNPCA: Congenital non-progressive cerebellar ataxia; EOA: early onset ataxia; FIHP: Familial Isolated Primary Hyperparathyroidism; GS: Gillespie Syndrome; R/C: Regulatory and Coupling Domain; SCA: Spinocerebellar Ataxia.

[a] Mutations reported in the reference sequence of NP_001093422 (IP_3R1), NP_002214 (IP_3R2), or NP_002215 (IP_3R3). If not originally reported in this reference sequence, multiple sequence alignment was utilized to find residue in appropriate reference sequence.

[b] This indicates the residue, base change, and reference sequence in which the mutation was originally discovered.

[c] This refers to whether residue is conserved among human, rat, and mouse sequences of all three IP_3R isoforms.

Mutation[a]	Original Residue (Base Change) & Reference Sequence[b]	Isoform	Domain	Conserved[c]	Diagnosis	Individuals Affected	Reference
L2403P	L2403P (c.7208T>C) in NM_001099952.2	*Itpr1*	1st-4th TM Domains	Yes	EOA	1 de novo case	Synofzik *et al.* 2018
S2454F	S2439F[d]	*Itpr1*	5th-6th TM Domains	No	SS	1 Individual	Prasad *et al.* 2016
T2490M	T2523M (c.7568C>T)[e]	*Itpr1*	5th-6th TM Domains	Yes	Unassigned SCA (Progressive optic atrophy, ataxia, etc.)	1 Individual	Valencia *et al.* 2015
G2506R	G2506R (c.7516G>A) in NM_001099952.2 & G2539R (c.7615G>A/C) in NM_001168272.1	*Itpr1*	Selectivity Filter	Yes	GS (c.7615G>A)	5 de novo cases	McEntagart *et al.* 2016
					SCA29	1 de novo case<br>1 inherited case	Zambonin *et al.* 2017
					GS (c.7615G>C)	1 de novo case	McEntagart *et al.* 2016
G2498S	G2498S (c.7492G>A) in NM_002223.2	*Itpr2*	Selectivity Filter	Yes	Anhidrosis	5 Homozygous cases & 5 Heterozygous cases (inherited)	Klar *et al.* 2014
S2508L	S2508L (chr12:26553068G>A)	*Itpr2*	TM	No	SS	1 Individual	Prasad *et al.* 2016
V2541A	V2574A (c.7721T>C) in NM_001168272	*Itpr1*	6th TM Domain	Yes	Molecularly unassigned SCA	Mother & Daughter (inherited)	Hsiao *et al.* 2017
N2543I	N2576I (c.7727A>T) in NM_001168272.1	*Itpr1*	6th TM Domain	Yes	GS	1 de novo case	Dentici *et al.* 2017
G2547A	G2547A (chr3:4856819G>C)	*Itpr1*	6th TM Domain	Yes	SCA29	1 de novo case	Gonzaga-Jauregui *et al.* 2015
I2550N	I2550N (c.7649T>A) in NM_001099952.2	*Itpr1*	6th TM Domain	Yes	PCH	1 de novo case	Van Dijk *et al.* 2016
I2550T	I2550T (c.7649T>C) in NM_001099952.2	*Itpr1*	6th TM Domain	Yes	SCA29	2 sporadic individuals	Zambonin *et al.* 2017
T2552P	T2585P (c.7753C>A) in NM_001168272.1	*Itpr1*	6th TM Domain	Yes	MICPCH	1 de novo case	Hayashi *et al.* 2017
F2553L	F2553L (c.7659T>G) in NM_001099952.2	*Itpr1*	6th TM Domain	Yes	GS	1 de novo case	Gerber *et al.* 2016
K2563del.	K2563del. in NM_001099952.2 K2596del. (c.7786_7788delAAG) in NM_001168272.1	*Itpr1*	LNK Domain	Yes	GS	4 de novo cases	McEntagart *et al.* 2016<br>Gerber *et al.* 2016
					SCA29 with Aniridia	1 de novo individual	Zambonin *et al.* 2017
					GS	1 de novo case	Dentici *et al.* 2017
					EOA w/Aniridia	1 de novo case	Synofzik *et al.* 2018

					GS	1 de novo case	de Silva *et al.* 2018

Table S4. Molecular characteristics of disease-associated IP_3R mutations in the c-terminal channel domain.
Abbreviations: EOA: early onset ataxia; GS: Gillespie Syndrome; LNK: Linker Domain; MICPCH: microcephaly with pontine and cerebellar hypoplasia; PCH: pontocerebellar hypoplasia; SCA: Spinocerebellar Ataxia; SS: Sézary Syndrome; TM: Transmembrane.
[a] Mutations reported in the reference sequence of NP_001093422 (IP_3R1), NP_002214 (IP_3R2), or NP_002215 (IP_3R3). If not originally reported in this reference sequence, multiple sequence alignment was utilized to find residue in appropriate reference sequence.
[b] This indicates the residue, base change, and reference sequence in which the mutation was originally discovered.
[c] This refers to whether residue is conserved among human, rat, and mouse sequences of all three IP_3R isoforms.
[d] Residue was originally reported as S2439F in *itpr1* without reference sequence. Assumption was made that reference sequence was human (NP_002213.5).
[e] Reference sequence of residue was not reported. Based on multiple sequence alignment, reference sequence was assumed to be in NM_001168272.1 based on appropriate amino acid residue present.

	Primer Name	Primer	Silent Mutation Introduced
**Primer 1**	R269W rIP$_3$R1 Forward	GCAGCACGTCTTCCTACGTACAACCGGCTGGCAGTCAGCCACGTCG	SNaBI
**Primer 2**	R269W rIP$_3$R1 Reverse	CGACGTGGCTGACTGCCAGCCGGTTGTACGTAGGAAGACGTGCTGC	SNaBI
**Primer 3**	R269W hIP$_3$R1 Forward	CAGCACGTCTTCCTACGTACCACGGGCTGGCAGTCGGCCACATCTGCC	SNaBI
**Primer 4**	R269W hIP$_3$R1 Reverse	GGCAGATGTGGCCGACTGCCAGCCCGTGGTACGTAGGAAGACGTGCTG	SNaBI
**Primer 5**	N602D rIP$_3$R1 Forward	GCCCTGCTCCACAACGATCGAAAGCTCCTG	PvuI
**Primer 6**	N602D rIP$_3$R1 Reverse	CAGGAGCTTTCGATCGTTGTGGAGCAGGGC	PvuI
**Primer 7**	T594I hIP$_3$R1 Forward	GATGTGTTGGCTGAAGATATCATCACTGCCCTGCTCC	EcoRV
**Primer 8**	T594I hIP$_3$R1 Reverse	GGAGCAGGGCAGTGATGATATCTTCAGCCAACACATC	EcoRV
**Primer 9**	G2506R (G>A) hIP$_3$R1 Forward	GAGTCACGGGCTACGTAGCAGGGGTGGAGTAGGAGATG	SNaBI
**Primer 10**	G2506R (G>A) hIP$_3$R1 Reverse	CATCTCCTACTCCACCCCTGCTACGTAGCCCGTGACTC	SNaBI
**Primer 11**	G2506R (G>C) hIP$_3$R1 Forward	GAGTCACGGGCTACGTAGCCGGGGTGGAGTAGGAG	SNaBI
**Primer 12**	G2506R (G>C) hIP$_3$R1 Reverse	CTCCTACTCCACCCCGGCTACGTAGCCCGTGACTC	SNaBI
**Primer 13**	G2498S mIP$_3$R2 Forward	GGCCTCAGGAATGGATCCGGAGTTGGGGATGTGCTG	BamHI
**Primer 14**	G2498S mIP$_3$R2 Reverse	CAGCACATCCCCAACTCCGGATCCATTCCTGAGGCC	BamHI

Table S5. Mutagenesis primers.
Primers used to introduce desired mutation and silently introduce restriction site.

Residue of Interest	Arg269	Thr594	Asn602	Gly2506	Gly2498
Original Sequence	*NP_001007236*	*NP_001093422*	*NP_001007236*	*NP_064307*	*NP_001093422*
NP_001093422 (hIP$_3$R1 variant 1)	269	594	602	2506	2507
NP_002213 (hIP$_3$R1 variant 2)	269	579	587	2491	2492
NP_001161744 (hIP$_3$R1 variant 3)	269	579	587	2539	2540
NP_001007236 (rIP$_3$R1 variant 1)	269	594	602	2544	2545
NP_001257525 (rIP$_3$R1 variant 2)	269	579	587	2529	2530
NP_001257526 (rIP$_3$R1 variant 3)	269	594	602	2505	2506
NP_034715 (mIP$_3$R1)	269	594	602	2545	2546
Q14643 (hIP$_3$R1 Structure)	269	594	602	2546	2547
P29994 (rIP$_3$R1 Structure)	269	594	602	2554	2555
NP_002214 (hIP$_3$R2)	269	594	602	2497	2498
NP_112308 (rIP$_3$R2)	269	594	602	2497	2498
NP_064307 (mIP$_3$R2 variant 1)	269	594	602	2497	2498
NP_34716 (mIP$_3$R2 variant 2)	235	561	569	2464	2465
NP_002215 (hIP$_3$R3)	270	594	602	2473	2474
NP_037270 (rIP$_3$R3)	270	594	602	2472	2473
NP_542120 (mIP$_3$R3)	270	594	602	2472	2473

Table S6. Corresponding residues in the other commonly used IP_3R sequences.
Residues of interest are referred to in the sequence indicated and the corresponding amino acid number is listed for other human, rat, and mouse sequences of all three IP_3R isoforms.

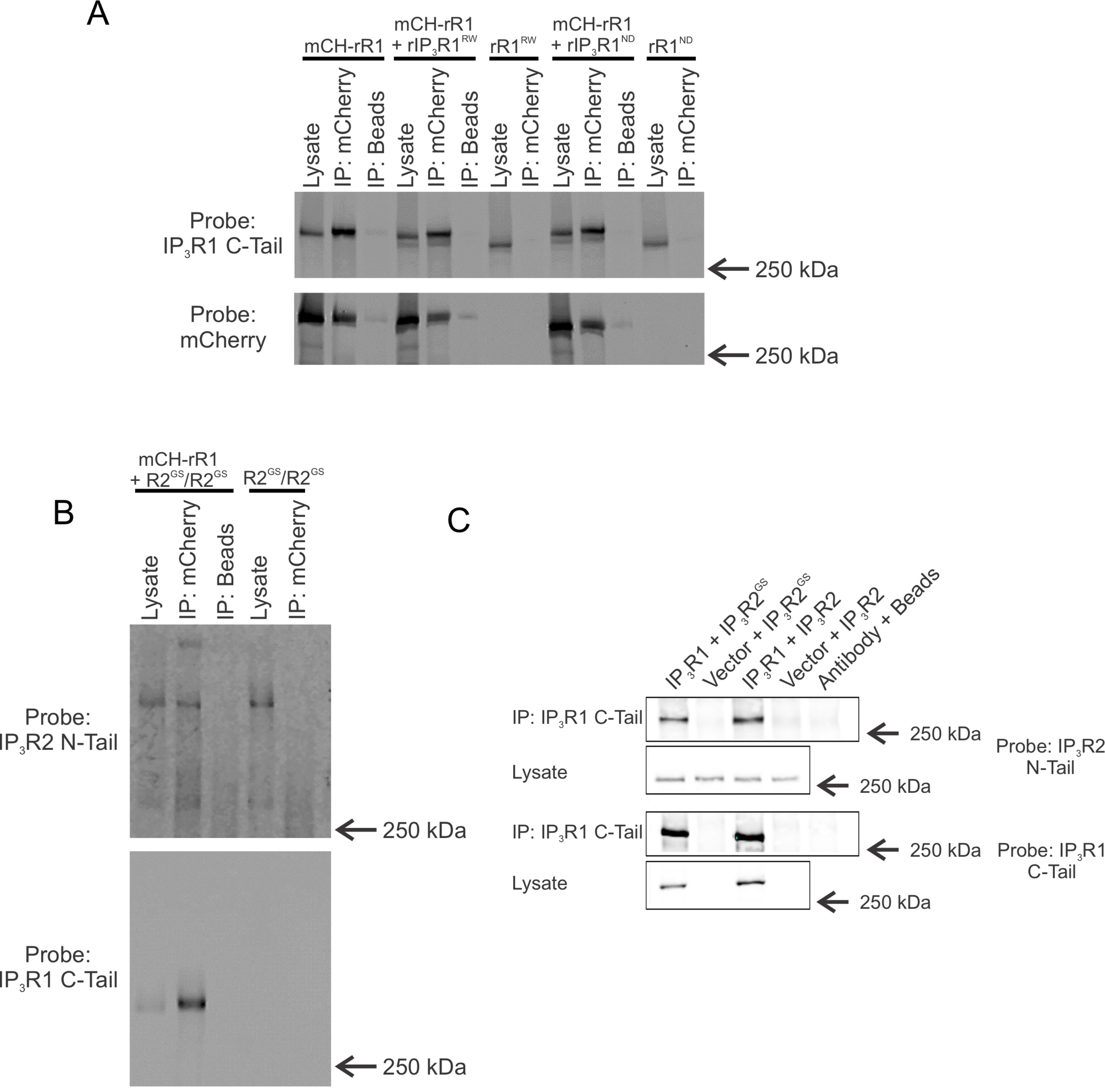

A
mCH-rR1
mCH-rR1 + rIP3R1 RW
rR1 RW
mCH-rR1 + rIP3R1 ND
rR1 ND
Lysate
IP: mCherry
IP: Beads
Probe: IP3R1 C-Tail
Probe: mCherry
250 kDa
B
mCH-rR1 + R2GS/R2GS
R2GS/R2GS
Probe: IP3R2 N-Tail
Probe: IP3R1 C-Tail
250 kDa
C
IP3R1 + IP3R2GS
Vector + IP3R2GS
IP3R1 + IP3R2
Vector + IP3R2
Antibody + Beads
IP: IP3R1 C-Tail
Lysate
250 kDa
Probe: IP3R2 N-Tail
Probe: IP3R1 C-Tail

Figure S1. Mutant IP_3R immunoprecipitated with wild-type IP_3R. **A.** mCherry tagged IP_3R1 was transiently co-transfected into HEK-3KO cells with or without rIP_3R1^{RW} and rIP_3R1^{ND}. Pulldown with mCherry antibody resulted in co-immunoprecipitation of both rIP_3R1^{RW} and rIP_3R1^{ND} when probed with c-terminal IP_3R1. **B.** $R2^{GS}/R2^{GS}$ was transiently co-transfected into HEK-3KO cells with or without mCherry tagged IP_3R1. Pulldown with an antibody for mCherry resulted in co-immunoprecipitation of $R2^{GS}/R2^{GS}$ when probed with n-terminal IP_3R2. **C.** WT mIP_3R2 and mIP_3R2^{GS} were transiently co-transfected into HEK-3KO cells with either rIP_3R1 or vector. Pulldown with α-c-terminal IP_3R1 resulted in co-immunoprecipitation of both WT mIP_3R2 and mIP_3R2^{GS} when probed with n-terminal IP_3R2.

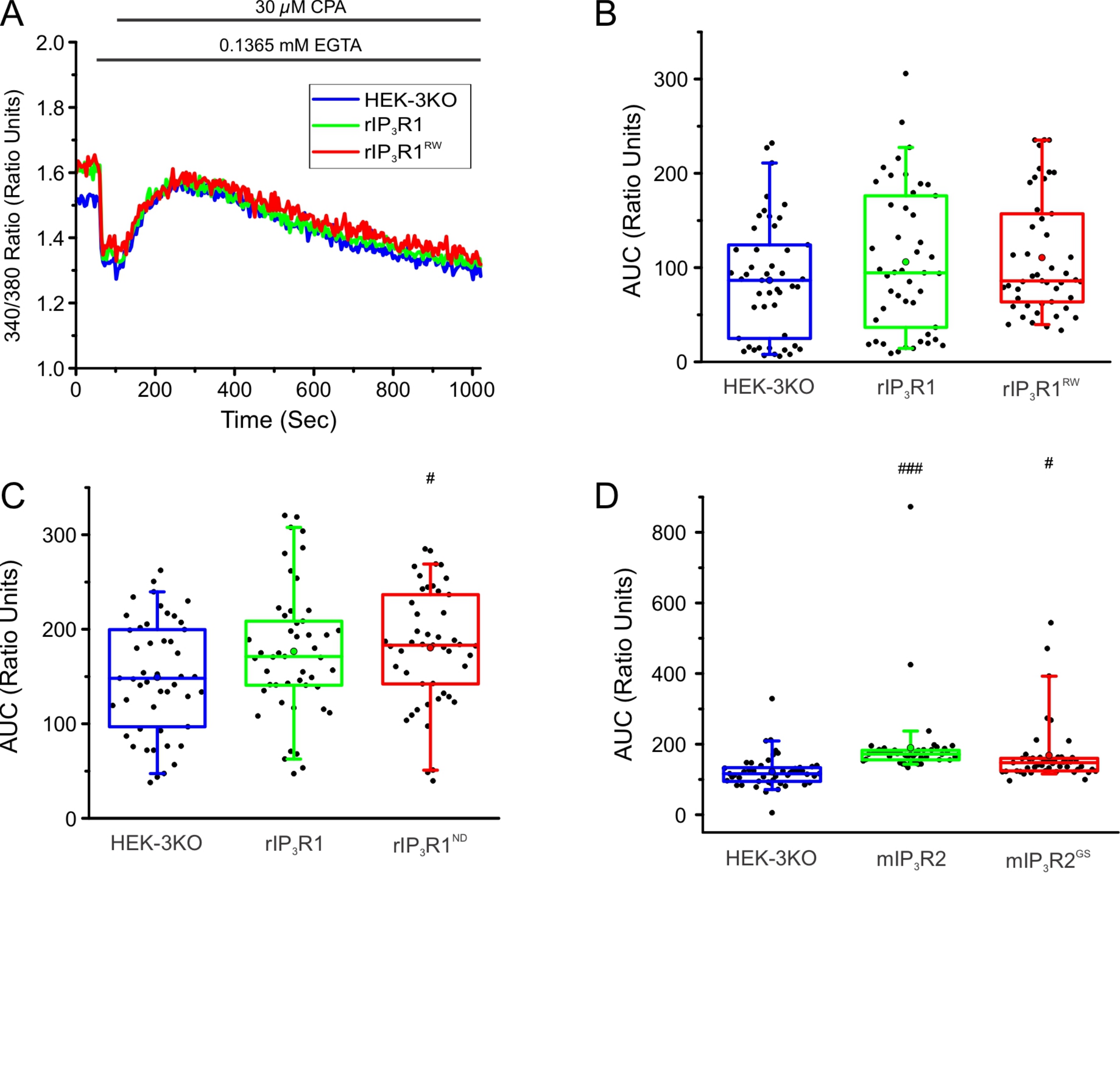
A
30 μM CPA
0.1365 mM EGTA
HEK-3KO
rIP3R1
rIP3R1RW
340/380 Ratio (Ratio Units)
Time (Sec)
B
AUC (Ratio Units)
HEK-3KO
rIP3R1
rIP3R1RW
C
#
AUC (Ratio Units)
HEK-3KO
rIP3R1
rIP3R1ND
D
###
#
AUC (Ratio Units)
HEK-3KO
mIP3R2
mIP3R2GS

S2

Figure S2. Stable expression of IP_3R mutants tested do not alter ER Ca^{2+} store content. **A.** HEK-3KO cells were loaded with Fura-2/AM and dispensed in a 96-well plate (~ 300,000 cells/well) in Ca^{2+} free imaging buffer. Fluorescence imaging was carried out using FlexStation 3 from Molecular Devices (excitation alternated between 340 nm and 380 nm and emission 510 nm). To force an extracellular Ca^{2+} concentration of 200nM, 0.1365 mM EGTA was added to the wells. Subsequently, 30 µM CPA was added to block SERCA and allow for emptying of the ER Ca^{2+} store. Results were exported to Excel where ratio of 340/380 values and area under the curve were calculated. **B.** Scatter plots summarizing Ca^{2+} content of ER store (area under the curve) for stable HEK-3KO cell lines expressing rIP_3R1^{RW}. Experiments were performed as shown in A. Boxes represent the 25th, 50th, and 75th percentiles, while whiskers represent 5th and 95th percentiles and mean is represented by colored circle. **C.** Scatter plots summarizing Ca^{2+} content of ER store (area under the curve) for stable HEK-3KO cell lines expressing rIP_3R1^{ND}. Experiments were performed as shown in A. Boxes represent the 25th, 50th, and 75th percentiles, while whiskers represent 5th and 95th percentiles and mean is represented by colored circle. **D.** Scatter plots summarizing Ca^{2+} content of ER store (area under the curve) for stable HEK-3KO cell lines expressing mIP_3R2^{GS}. Experiments were performed as shown in A. Boxes represent the 25th, 50th, and 75th percentiles, while whiskers represent 5th and 95th percentiles and mean is represented by colored circle. Unless otherwise stated, all data above comes from at least N=3 experiments. $^{\#}P < 0.05$ and $^{\#\#\#}P < 0.001$ when compared to HEK-3KO cell line; one-way ANOVA with Tukey's test was performed in B ($F_{2,91} = 1.630, p < 0.2017$), C ($F_{2,140} = 3.408, p < 0.0359$), and D ($F_{2,141} = 8.002, p < 0.0005$).

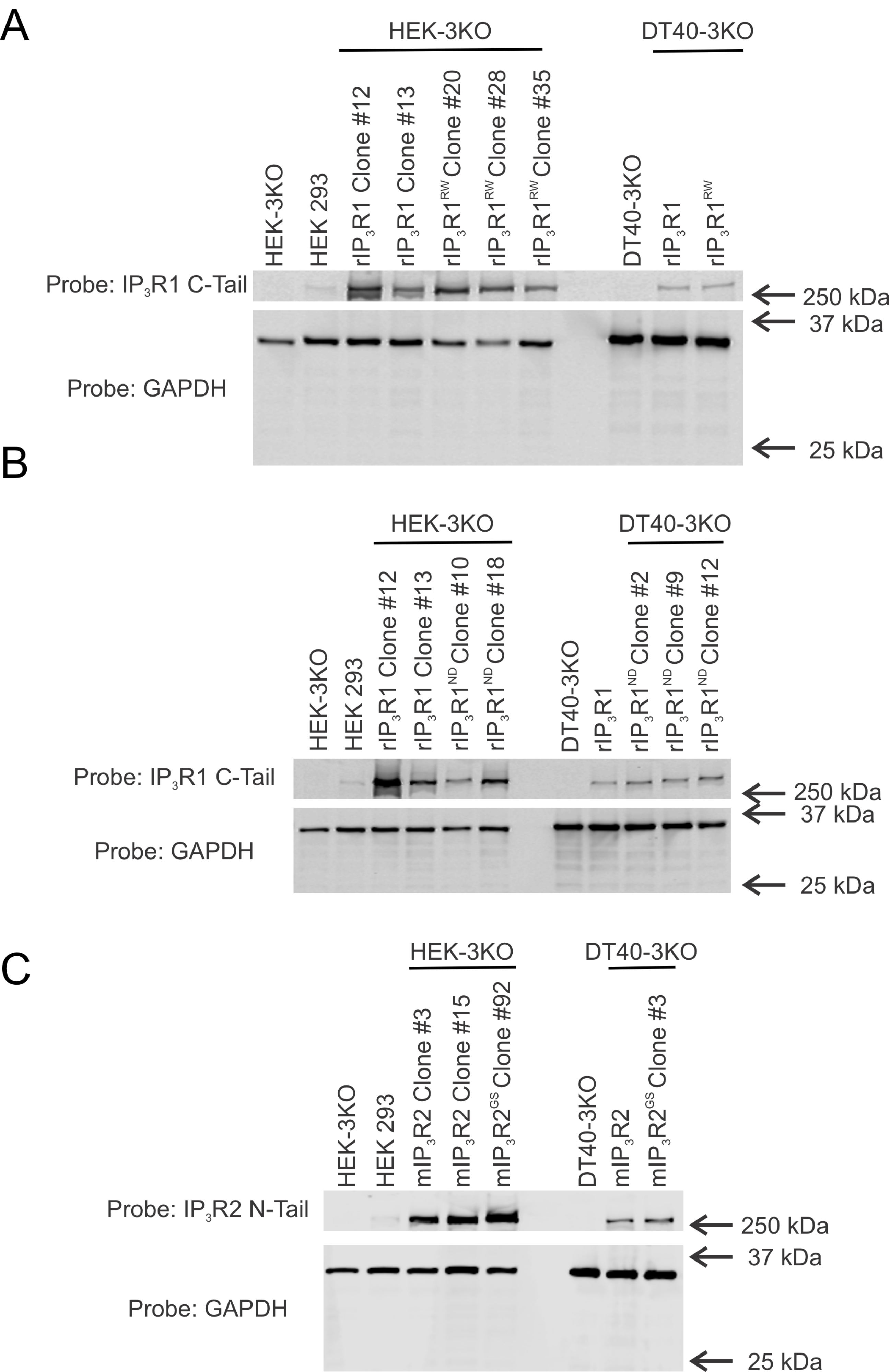
A
HEK-3KO
DT40-3KO
HEK-3KO
HEK 293
rIP3R1 Clone #12
rIP3R1 Clone #13
rIP3R1RW Clone #20
rIP3R1RW Clone #28
rIP3R1RW Clone #35
DT40-3KO
rIP3R1
rIP3R1RW
Probe: IP3R1 C-Tail
250 kDa
37 kDa
Probe: GAPDH
25 kDa
B
HEK-3KO
DT40-3KO
HEK-3KO
HEK 293
rIP3R1 Clone #12
rIP3R1 Clone #13
rIP3R1ND Clone #10
rIP3R1ND Clone #18
DT40-3KO
rIP3R1
rIP3R1ND Clone #2
rIP3R1ND Clone #9
rIP3R1ND Clone #12
Probe: IP3R1 C-Tail
250 kDa
37 kDa
Probe: GAPDH
25 kDa
C
HEK-3KO
DT40-3KO
HEK-3KO
HEK 293
mIP3R2 Clone #3
mIP3R2 Clone #15
mIP3R2GS Clone #92
DT40-3KO
mIP3R2
mIP3R2GS Clone #3
Probe: IP3R2 N-Tail
250 kDa
37 kDa
Probe: GAPDH
25 kDa

Figure S3. Mutant expression is increased in HEK-3KO cells compared to DT40-3KO cells. **A.** WT rIP_3R1 and mutant rIP_3R1^{RW} cell lines were generated in the IP_3R-null HEK-3KO cells and DT40-3KO cells and western blotted for c-terminal IP_3R1 and GAPDH. **B.** WT rIP_3R1 and mutant rIP_3R1^{ND} cell lines were generated in the IP_3R-null HEK-3KO cells and DT40-3KO cells and western blotted for c-terminal IP_3R1 and GAPDH. **C.** WT mIP_3R2 and mutant mIP_3R2^{GS} cell lines were generated in the IP_3R-null HEK-3KO cells and DT40-3KO cells and western blotted for n-terminal IP_3R2 and GAPDH.

A

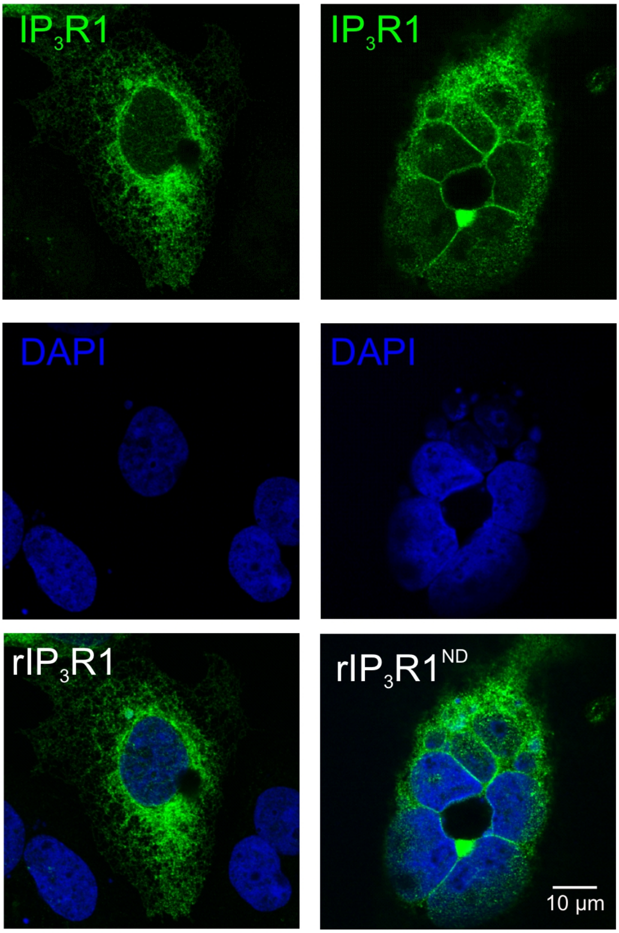
IP3R1
IP3R1
DAPI
DAPI
rIP3R1
rIP3R1ND
10 µm

B

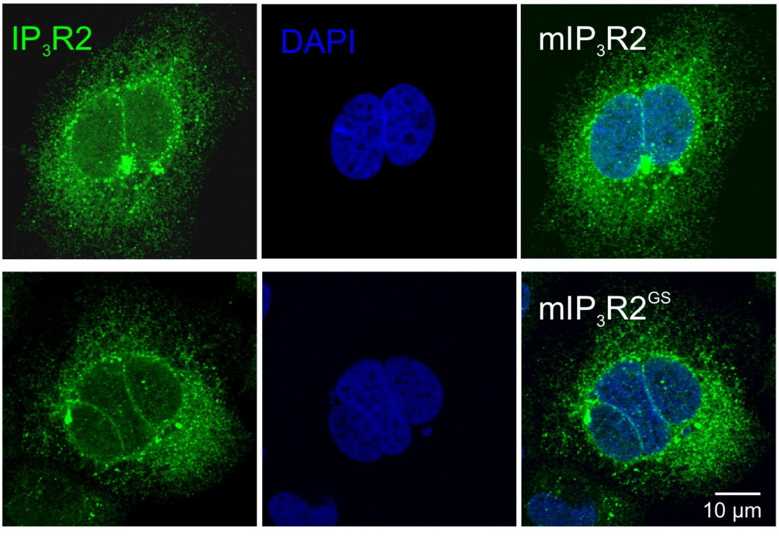
IP3R2
DAPI
mIP3R2
mIP3R2GS
10 µm

Figure S4. rIP_3R1^{ND} and mIP_3R2^{GS} constructs localize to the ER membrane when stably expressed in HEK-3KO cells. **A.** Immunocytochemistry for HEK-3KO cell lines expressing either WT rIP_3R1 (left) or mutant rIP_3R1^{ND} (right). Top, IP_3R1 detection (green); middle, DAPI detection (blue); bottom, merged images of IP_3R1 and DAPI. Scale bars, 10 μm. **B.** Immunocytochemistry for HEK-3KO cell lines expressing either WT mIP_3R2 (top) or mutant mIP_3R2^{GS} (bottom). Left, IP_3R2 detection (green); middle, DAPI detection (blue); right, merged images of IP_3R2 and DAPI. Scale bars, 10 μm.

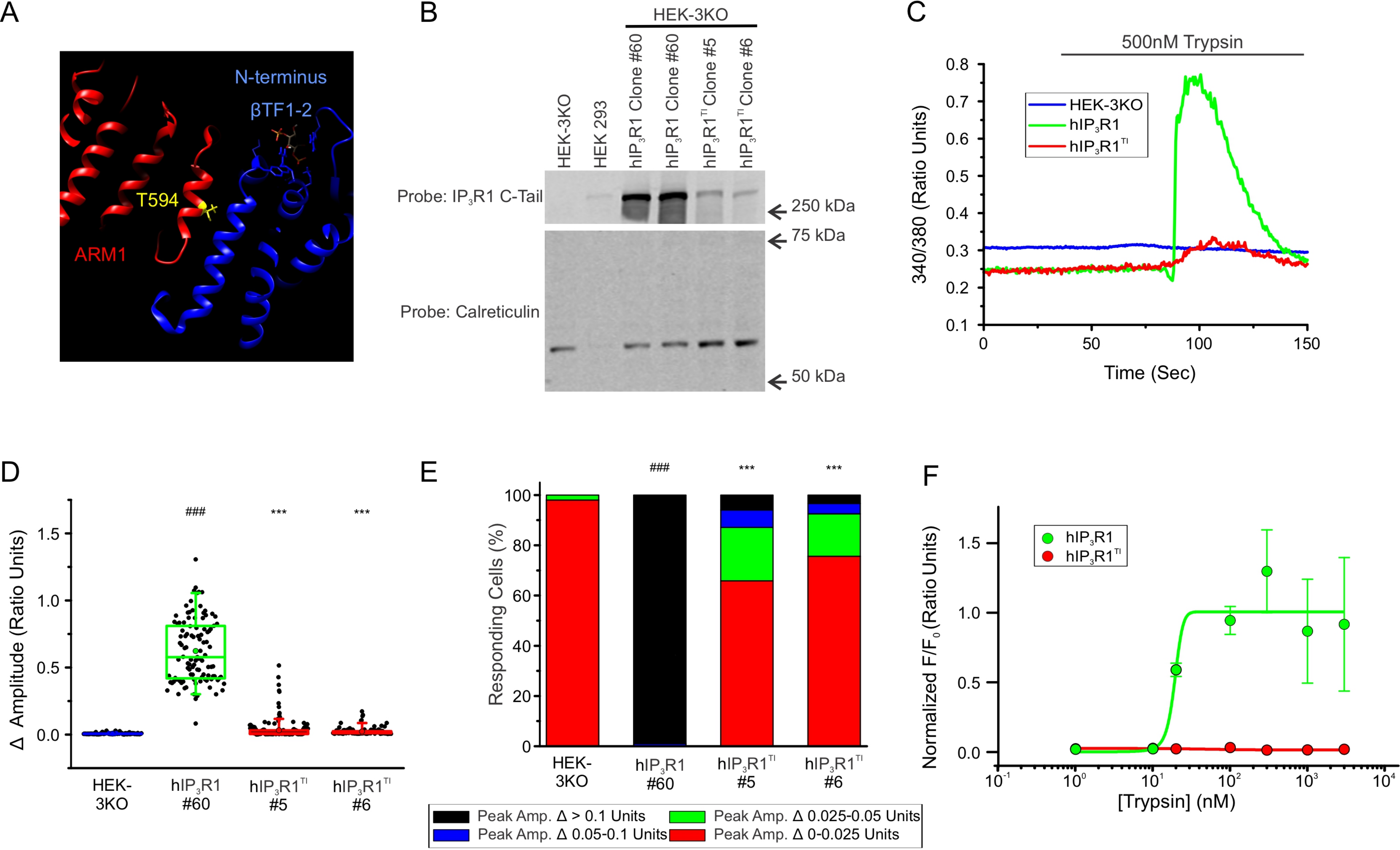
A
N-terminus
βTF1-2
T594
ARM1
B
HEK-3KO
HEK-3KO
HEK 293
hIP3R1 Clone #60
hIP3R1 Clone #60
hIP3R1TI Clone #5
hIP3R1TI Clone #6
Probe: IP3R1 C-Tail
Probe: Calreticulin
250 kDa
75 kDa
50 kDa
C
500nM Trypsin
HEK-3KO
hIP3R1
hIP3R1TI
340/380 (Ratio Units)
0.1
0.2
0.3
0.4
0.5
0.6
0.7
0.8
0
50
100
150
Time (Sec)
D
Δ Amplitude (Ratio Units)
0.0
0.5
1.0
1.5
###
***
***
HEK-3KO
hIP3R1 #60
hIP3R1TI #5
hIP3R1TI #6
E
Responding Cells (%)
0
20
40
60
80
100
###
***
***
HEK-3KO
hIP3R1 #60
hIP3R1TI #5
hIP3R1TI #6
Peak Amp. Δ > 0.1 Units
Peak Amp. Δ 0.05-0.1 Units
Peak Amp. Δ 0.025-0.05 Units
Peak Amp. Δ 0-0.025 Units
F
hIP3R1
hIP3R1TI
Normalized F/F0 (Ratio Units)
0.0
0.5
1.0
1.5
10-1
100
101
102
103
104
[Trypsin] (nM)

S5

Figure S5. rIP_3R1^{TI} is poorly functional when expressed in HEK-3KO cells. **A.** Chimera (PDB: 6DQN) was used to visualize WT Thr594 (yellow) located near the junction of the ARM1 domain (red) and the β-TF1 and β-TF2 domains of the LBD in the N-terminus (blue). **B.** WT hIP_3R1 and multiple mutant hIP_3R1^{TI} cell lines generated in the IP_3R-null HEK-3KO cells were western blotted. **C.** Representative traces show Ca^{2+} signals of IP_3R-null HEK-3KO cells (blue), WT hIP_3R1 (green), and hIP_3R1^{TI} (red) in response to trypsin (500 nM) when loaded with Fura-2/AM. **D.** Scatter plots summarizing change in amplitude (Peak ratio – Basal ratio: average of initial 5 ratio points) for experiments similar to those shown in B when treated with 500 nM trypsin. Boxes represent the 25th, 50th, and 75th percentiles, while whiskers represent 5th and 95th percentiles and mean is represented by colored circle. **E.** Stacked bar graph summarizing the percentage of amplitudes from D which fall into pre-determined ranges such that only those cells with an amplitude change greater than 0.1 ratio units (black portion of bars) are considered to be responding to the trypsin stimulus shown in C. **F.** Dose-response curve showing Ca^{2+} response of Fura-2/AM loaded WT hIP_3R1 and hIP_3R1^{TI} cells when treated with increasing concentrations (1 nM, 10 nM, 30 nM, 100 nM, 300 nM, 1 μM, and 3 μM) of trypsin using a Flexstation3 96-well plate reader. Data are mean ± SEM of three (N = 3) independent experiments. $^{***}P < 0.001$ when compared to WT rIP_3R1 cell line and $^{\#\#\#}P < 0.001$ when compared to HEK-3KO cell line; one-way ANOVA with Tukey's test was performed in D ($F_{3,530} = 784.8$, $p < 0.0001$) and E ($F_{3,10} = 298.7$, $p < 0.0001$). Unless otherwise stated, all data above comes from at least N=3 experiments.

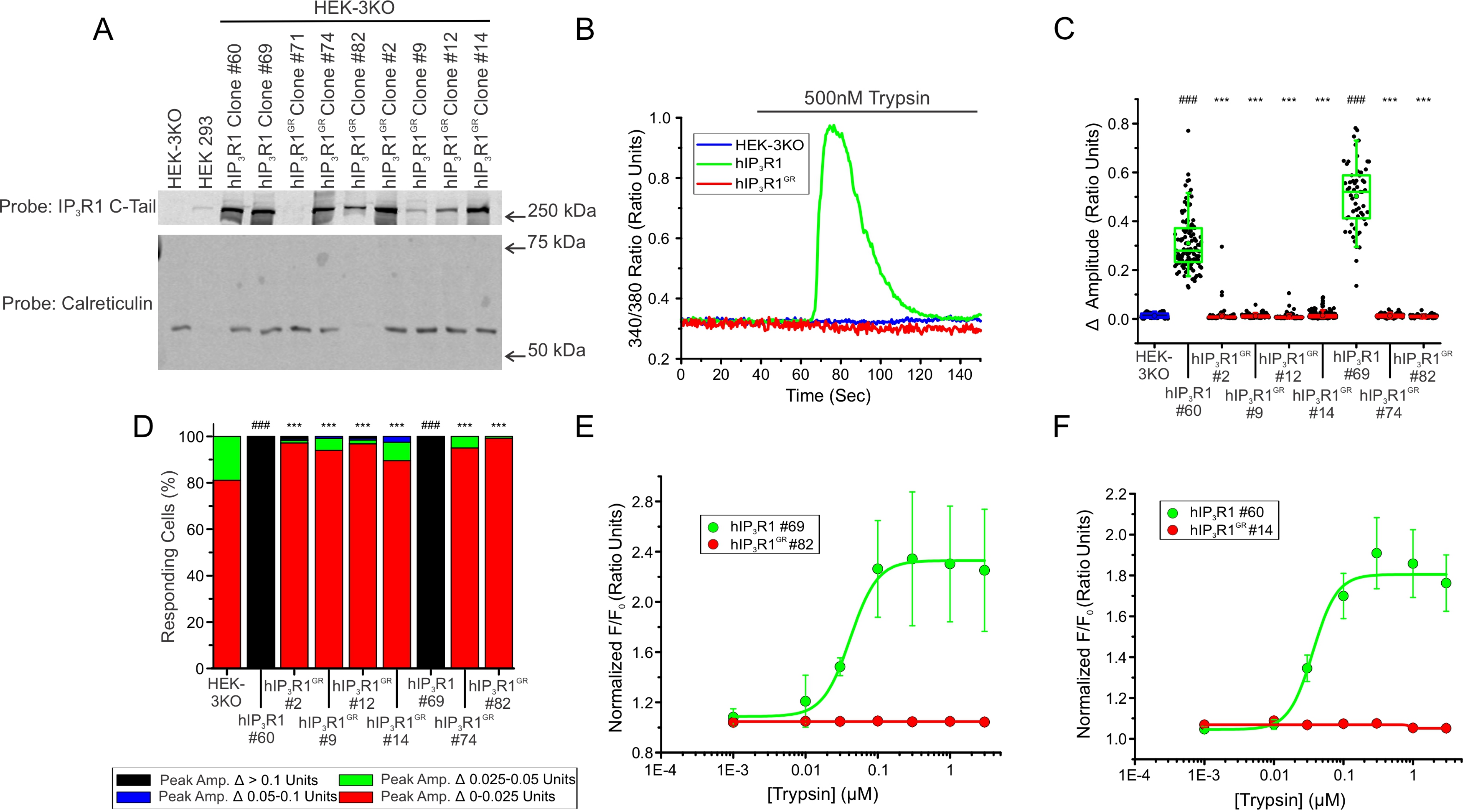
A
HEK-3KO
HEK-3KO
HEK 293
hIP3R1 Clone #60
hIP3R1 Clone #69
hIP3R1GR Clone #71
hIP3R1GR Clone #74
hIP3R1GR Clone #82
hIP3R1GR Clone #2
hIP3R1GR Clone #9
hIP3R1GR Clone #12
hIP3R1GR Clone #14
Probe: IP3R1 C-Tail
Probe: Calreticulin
250 kDa
75 kDa
50 kDa
B
500nM Trypsin
HEK-3KO
hIP3R1
hIP3R1GR
340/380 Ratio (Ratio Units)
Time (Sec)
C
Δ Amplitude (Ratio Units)
*** *** *** *** ### *** ***
HEK-3KO
hIP3R1 #60
hIP3R1GR #2
hIP3R1GR #9
hIP3R1GR #12
hIP3R1GR #14
hIP3R1 #69
hIP3R1GR #74
hIP3R1GR #82
D
Responding Cells (%)
*** *** *** *** ### *** ***
Peak Amp. Δ > 0.1 Units
Peak Amp. Δ 0.05-0.1 Units
Peak Amp. Δ 0.025-0.05 Units
Peak Amp. Δ 0-0.025 Units
E
hIP3R1 #69
hIP3R1GR #82
Normalized F/F0 (Ratio Units)
[Trypsin] (μM)
F
hIP3R1 #60
hIP3R1GR #14
Normalized F/F0 (Ratio Units)
[Trypsin] (μM)

Figure S6. hIP_3R1^{GR} is non-functional when expressed in HEK-3KO cells. **A.** Multiple WT hIP_3R1 and mutant hIP_3R1^{GR} cell lines were generated in the IP_3R-null HEK-3KO cells and western blotted. hIP_3R1^{GR} cell lines #2, #9, #12, and #14 contain the GGG to CGG mutation made using primers 9 and 10 (Table S5), while hIP_3R1^{GR} cell lines #74 and #82 contain the GGG to AGG mutation made using primers 7 and 8 (Table S5). **B.** Representative traces show Ca^{2+} signals of IP_3R-null HEK-3KO cells (blue), WT hIP_3R1 (green), and hIP_3R1^{GR} (red) in response to trypsin (500 nM) when loaded with Fura-2/AM. **C.** Scatter plots summarizing change in amplitude (Peak ratio – Basal ratio: average of initial 5 ratio points) for experiments similar to those shown in B when treated with 5 nM, 50nM, and 500 nM of trypsin. Boxes represent the 25th, 50th, and 75th percentiles, while whiskers represent 5th and 95th percentiles and mean is represented by colored circle. **D.** Stacked bar graph summarizing the percentage of amplitudes from C which fall into pre-determined ranges such that only those cells with an amplitude change greater than 0.1 ratio units (black portion of bars) are considered to be responding to the trypsin stimulus shown in B. **E.** Dose-response curve showing Ca^{2+} response of Fura-2/AM loaded WT hIP_3R1 cell line #60 and hIP_3R1^{GR} cell line #14 when treated with increasing concentrations (1 nM, 10 nM, 30 nM, 100 nM, 300 nM, 1 μM, and 3 μM) of trypsin using a Flexstation3 96-well plate reader. Data are mean ± SEM of three (N = 3) independent experiments. **F.** Dose-response curve showing Ca^{2+} response of Fura-2/AM loaded WT hIP_3R1 cell line #69 and hIP_3R1^{GR} cell line #82 when treated with increasing concentrations (1 nM, 10 nM, 30 nM, 100 nM, 300 nM, 1 μM, and 3 μM) of trypsin using a Flexstation3 96-well plate reader. Data are mean ± SEM of three (N = 3) independent experiments. $^{***}P < 0.001$ when compared to WT rIP_3R1 cell line and $^{\#\#\#}P < 0.001$ when compared to HEK-3KO cell line; one-way ANOVA with Tukey's test was performed in C ($F_{8,1264} = 1245$, $p < 0.0001$) and D ($F_{8,28} = 16203$, $p < 0.0001$). Unless otherwise stated, all data above comes from at least N=3 experiments.
